# Neuron-specific ablation of the Krabbe disease gene galactosylceramidase in mice results in neurodegeneration

**DOI:** 10.1371/journal.pbio.3001661

**Published:** 2022-07-05

**Authors:** Conlan Kreher, Jacob Favret, Nadav I. Weinstock, Malabika Maulik, Xinying Hong, Michael H. Gelb, Lawrence Wrabetz, M. Laura Feltri, Daesung Shin

**Affiliations:** 1 Institute for Myelin and Glia Exploration, Jacobs School of Medicine and Biomedical Sciences, University at Buffalo—SUNY, Buffalo, New York, United States of America; 2 Department of Biotechnical and Clinical Laboratory Sciences, Jacobs School of Medicine and Biomedical Sciences, University at Buffalo—SUNY, Buffalo, New York, United States of America; 3 Department of Neurology, Jacobs School of Medicine and Biomedical Sciences, University at Buffalo—SUNY, Buffalo, New York, United States of America; 4 Departments of Chemistry and Biochemistry, University of Washington, Seattle, Washington, United States of America; 5 Department of Biochemistry, Jacobs School of Medicine and Biomedical Sciences, University at Buffalo—SUNY, Buffalo, New York, United States of America; Oregon Health and Science University, UNITED STATES

## Abstract

Krabbe disease is caused by a deficiency of the lysosomal galactosylceramidase (GALC) enzyme, which results in the accumulation of galactosylceramide (GalCer) and psychosine. In Krabbe disease, the brunt of demyelination and neurodegeneration is believed to result from the dysfunction of myelinating glia. Recent studies have shown that neuronal axons are both structurally and functionally compromised in Krabbe disease, even before demyelination, suggesting a possible neuron-autonomous role of GALC. Using a novel neuron-specific *Galc* knockout (CKO) model, we show that neuronal *Galc* deletion is sufficient to cause growth and motor coordination defects and inflammatory gliosis in mice. Furthermore, psychosine accumulates significantly in the nervous system of neuron-specific *Galc*-CKO. Confocal and electron microscopic analyses show profound neuro-axonal degeneration with a mild effect on myelin structure. Thus, we prove for the first time that neuronal GALC is essential to maintain and protect neuronal function independently of myelin and may directly contribute to the pathogenesis of Krabbe disease.

## Abstract 2

Krabbe disease is a demyelinating neurodegenerative disorder caused by a deficiency of the enzyme lysosomal galactosylceramidase (GALC), which results in the accumulation of galactosylceramide and psychosine. This study uses a novel neuron-specific knockout model in the first in vivo attempt to investigate the role of neuronal GALC in neuronal function and the etiology of Krabbe disease.

## Introduction

Krabbe disease is caused by a deficiency of galactosylceramidase (GALC) [1], a lysosomal acid hydrolase that catabolizes the lipids galactosylceramide (GalCer) and galactosylsphingosine (= psychosine). A recent study suggests that acid ceramidase can deacylate GalCer into psychosine [2,3]. In Krabbe tissues, especially within the brain [4], psychosine accumulates to high levels, triggering membrane destabilization and cell death [5–9]. Other pathologic hallmarks of Krabbe disease include rapid and progressive demyelination of the central and peripheral nervous systems (CNS and PNS, respectively) with diffuse Periodic Acid–Schiff positive multinucleated macrophage infiltration [10] and reactive astrogliosis [11–13].

Since Krabbe disease begins during active myelination and is characterized by extensive demyelination with relative sparing of the gray matter [14], oligodendrocytes (OLs) have been regarded as the first and primary cells targeted by the pathology (reviewed in [15]). This demyelination should secondarily affect neurons, whose degeneration likely contributes significantly to major symptoms of the disease. While myelinating glia have traditionally been considered as primary drivers of Krabbe pathogenesis and the cause for most neurological symptoms in Krabbe patients, the extent and contribution for other cell types including neurons remain unknown. In fact, GALC is expressed ubiquitously and peaks at an early developmental period in the brains of postnatal mice [16–19], indicating a possibility that other brain cells could contribute to the disease. Indeed, evidence suggests that cell types other than OLs could contribute to Krabbe disease. For example, in an in vitro system, cultured axons become primarily and progressively dysfunctional in response to psychosine, causing lipid raft clustering and finally generating a dying-back neuropathy [16,20]. Induced neurons from Krabbe patient’s fibroblasts had axonal defects that might be caused by neuron-autonomous psychosine accumulation [21], yet whether the neuroaxonal pathology was neuron-autonomous remains unclear due to the ubiquitous nature of GALC. It was possible cultured neurons were already conditioned by toxicities from other cell types. Therefore, we previously used *Thy1Cre/ER*^*T2*^-mediated neuron-specific *Galc* knockout (KO) mouse model, to study the neuron-autonomous function of GALC without other cells’ effects [17]. Interestingly, we found that immature neurons are increased in the brain of neuron-specific mutants when GALC is depleted before developmental myelination starts in mice. However, this induced neuron-specific *Galc* KO mouse did not show any pathological symptoms including psychosine accumulation. We hypothesized that this might be due to the inability of *Thy1Cre/ER*^*T2*^ mice to have sufficient neuronal expression.

To this end, to maximize the ablation effect of *Galc* in neurons at all times, in this study, we generated pan-neuronal *Galc* KO mice using a *Galc-flox* allele [17] crossed with *Syn1Cre* substrain that deletes *Galc* gene constitutively [22,23]. As expected, the Cre activity was quite robust in most neurons and had a neuropathological phenotype that was not seen in the previous *Thy1Cre/ER*^*T2*^-mediated *Galc* KO mouse. This model allowed us for the first time in an in vivo system to investigate the neuron-autonomous role of GALC and, furthermore, the effects of neuronal GALC ablation on motor function, biochemical changes, and morphological abnormalities in Krabbe disease. Notably, we showed a distinct neuro-axonal degeneration with a marginal effect on myelin structure, implying a neuron-autonomous GALC function.

## Results

### *Galc* is efficiently deleted in the neurons of *Syn1Cre*-mediated *Galc* KO mice

**Fig 1:**
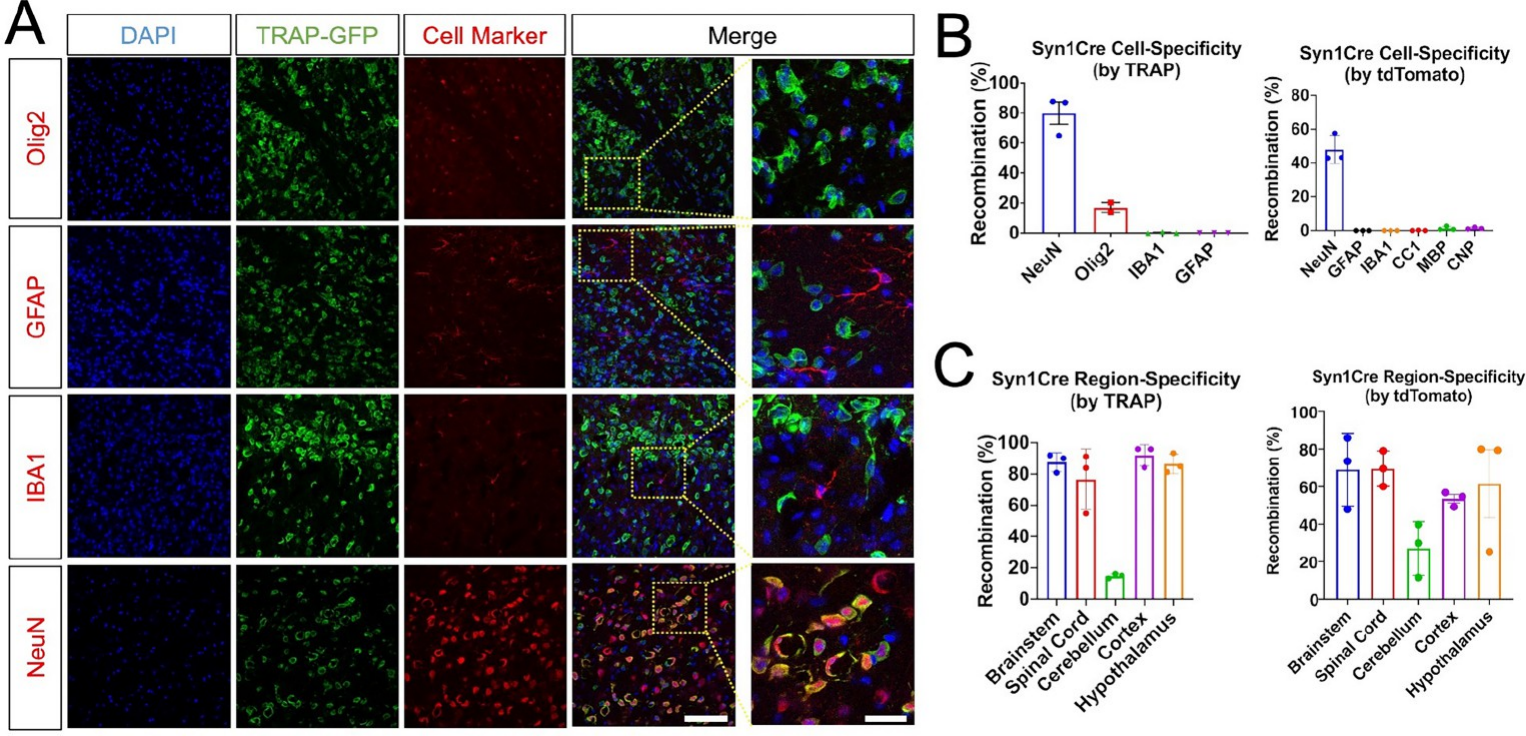
Syn1Cre is specific to neurons. (**A**) *Syn1Cre* mice were crossed with EGFP/L10a reporter (TRAP) mice, to study Cre recombination efficiency. Immunohistochemistry on P15 brain shows that TRAP-GFP is expressed mostly in neurons (NeuN) and far higher than the expression in OLs (Olig2), microglia (Iba1), or astrocytes (GFAP). Representative images are from cerebral cortex. Scale bars = 100 μm and 30 μm, respectively. DAPI is blue-colored. (**B**) Quantification of colocalized TRAP-GFP in each cell type reveals that the majority of TRAP-GFP at P15 is expressed in neurons. Unexpectedly, approximately 15% of TRAP-GFP signals colocalized with Olig2, presumably due to the fact that Olig2 is also expressed in a subset of MNs [25]. To further confirm if *Syn1Cre* is indeed specific to neurons, we also tested *Syn1Cre* expression in another Cre-reporter system, tdTomato mice. The tdTomato was not colocalized with CC1, MBP, CNP, IBA1 nor GFAP, but only with NeuN, suggesting that *Syn1Cre* is predominantly expressed in neurons. (**C**) Quantifications of both colocalized TRAP-GFP and tdTomato in neurons of subanatomical regions shows that Syn1Cre activity is particularly high in the neurons of brainstem, spinal cord, cerebral cortex, and hypothalamus, whereas it is marginal in cerebellar neurons, consistent with the fact that granular neurons in the cerebellum do not express *Synapsin I* [22, 23]. Data are presented as mean values ± SEM. *N* = 3. The underlying data for **B** and **C** can be found in S1 Data. MN, motor neuron; OL, oligodendrocyte; TRAP, Translating Ribosome Affinity Purification.

To assess the role of GALC in neurons, and its relationship to the pathogenesis of Krabbe disease, we used a recently developed conditional *Galc* floxed mouse [17] and a pan-neuronal–specific *Syn1Cre* mouse line (JAX#003966) [22,23]. First, we confirmed if the *Syn1Cre* line is efficiently and specifically activated in neurons in our hands, although this Cre line is already characterized in the literature [23]. To examine the spatial pattern and cell specificity, we crossed the *Syn1Cre* mice with the reporter line named Translating Ribosome Affinity Purification (TRAP) expressing a GFP-tagged L10a ribosomal protein that is activated only in the presence of CRE [24]. To quantitatively determine the specificity of *Syn1Cre* activity, cryo-sectioned brains from 2-month-old *Syn1Cre; TRAP* mice were immunostained with cell type–specific markers Olig2 (OL lineage cells), GFAP (astrocytes), IBA1 (microglia), and NeuN (neurons). More than 80% of TRAP-GFP overlapped with the neuronal marker, but not at all with the marker for microglia or astrocytes. Unexpectedly, 15% to 20% of TRAP-GFP signals colocalized with a marker for OLs, Olig2 (**Figs 1A, 1B and** S1), presumably due to the fact that Olig2 is also expressed in a subset of motor neurons (MNs) [25]. To further confirm if *Syn1Cre* is indeed specific to neurons, we also tested *Syn1Cre* expression in a second Cre-reporter system, the tdTomato mouse (JAX#007905) [26]. The *Syn1Cre* line was crossed with the tdTomato mice and immunostained with alternative OL markers; CC1, MBP, and CNPase, along with other cell type markers. The tdTomato was not colocalized with CC1, MBP, CNPase, IBA1 nor GFAP, but only with NeuN (**Figs 1B** and S1B). This suggests that *Syn1Cre* is predominantly expressed in neurons as opposed to other glial cell types including OLs, although there is a possibility of rare cryptic recombination in nonneuronal cells that could be a limit of the *Cre-loxP* system. In addition, Syn1Cre was active in most brain regions and spanned the brainstem, cerebral cortex, hypothalamus, and spinal cord (**Fig 1C**). One noted caveat of our model system was the moderate expression of *Syn1Cre* in cerebellar neurons, likely related to the limited expression of *Synapsin1* in neurons of the cerebellum [22,23]. Taken together, these results suggest that *Syn1Cre* is specific and widely expressed in most neurons throughout the murine CNS.

**Fig 2:**
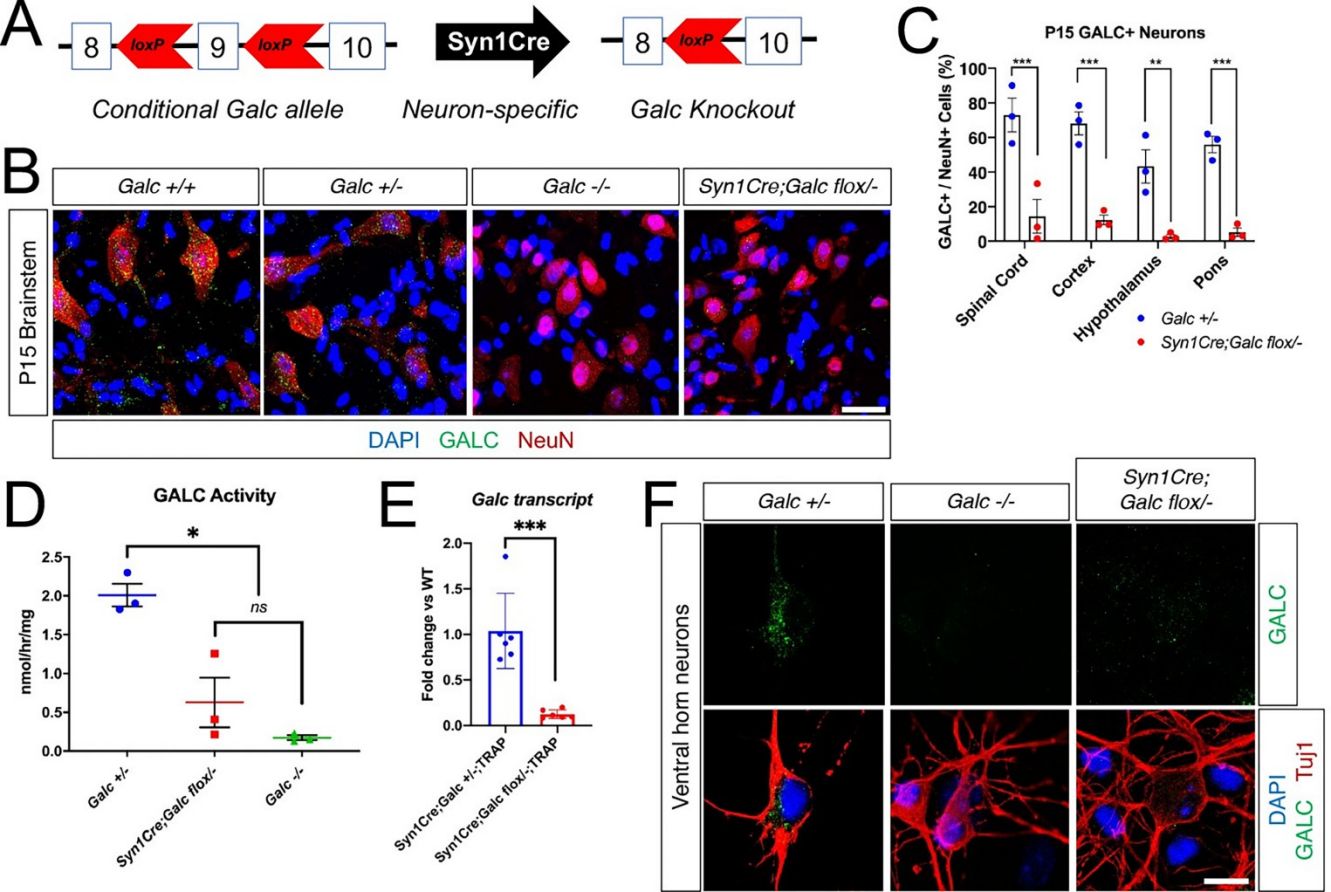
GALC protein is efficiently depleted in the neurons of *Syn1Cre; Galc flox/−*. (**A**) To delete *Galc* in neurons, *Galc*-floxed conditional allele mouse was crossed with *Syn1Cre* mouse. (**B**) Immunohistochemistry on P15 brain and spinal cord shows that GALC is dramatically reduced in the NeuN-positive neurons of *Syn1Cre; Galc flox/−*. Scale bar = 50 μm. (**C**) Quantification and comparison of GALC-positive cells in the brains of *Galc* heterozygotes (*Galc +/−*) and *Syn1Cre; Galc flox/−* reveals that GALC protein is efficiently depleted in the neurons of spinal cord, cerebral cortex, hypothalamus, and pons from the *Syn1Cre; Galc flox/−* mice. *N* = 3. Data are presented as mean values ± SEM; **P < 0.01, ***P < 0.001. (**D**) GALC enzymatic activities were measured in total brain lysates from 6-month-old animals. The GALC activity was significantly reduced in the *Syn1Cre; Galc flox/−* versus control (*Galc +/−*). *N* = 3. Data are presented as mean values ± SEM; *P < 0.05. (**E**) *Galc* transcripts were assessed by RT-qPCRs from total RNAs purified from ribosomes, which were pulled down from the brains of both *Syn1Cre; Galc flox/−; TRAP* and *Syn1Cre; Galc +/−; TRAP*. *Galc* was dramatically reduced in *Syn1Cre; Galc flox/−* brains, compared to *Syn1Cre; Galc +/−*. *N* = 6. Data are presented as mean values ± SD; ***P < 0.001. (**F**) Ventral horn neurons were purified from 12.5 to 13.5 embryos and cultured for 7 days. Immunocytochemistry shows that GALC (green) is much reduced in the neurons (Tuj1; red), compared to that of control (*Galc +/−*). Scale bar = 10 μm. The underlying data for **C**–**E** can be found in S1 Data. GALC, galactosylceramidase.

Next, to delete *Galc* in neurons, we crossed the *Syn1Cre* line with the *Galc-flox* mouse [17] (**Fig 2A**). To maximize the GALC depletion effect, we used the haplodeficient *Galc* heterozygote: *Syn1Cre; Galc flox/−*. To validate the reduction of GALC in the CNS of *Syn1Cre; Galc flox/−*, we immunostained the neurons and glia, along with GALC at P15 when the CNS is actively undergoing developmental myelination [27]. This analysis showed a significant reduction of the percentage of GALC-positive neurons in the CNS of the mutant compared to *Galc +/−* control (**Fig 2B and 2C**). Furthermore, GALC enzymatic activities were measured in total brain lysates from 6-month-old animals. GALC activity was significantly reduced in *Syn1Cre; Galc flox/−*, compared to control (*Galc +/−*) (**Fig 2D**). We next measured *Galc* transcripts from polysomes, which were purified from total brains of 2-month-old *Syn1Cre; Galc flox/−; TRAP*. The level of *Galc* transcripts was significantly reduced in the *Syn1Cre; Galc flox/−; TRAP* mice compared to control *Syn1Cre; Galc +/−; TRAP* (**Fig 2E**). In addition, GALC levels in cultured neurons from the spinal cord of *Syn1Cre; Galc flox/−* was dramatically reduced compared to that of controls (*Galc +/+* or *+/−*) (**Fig 2F**), suggesting a specific *Galc* deletion in the neurons of *Syn1Cre; Galc flox/−*.

### Neuron-specific *Galc* deletion causes a neurological phenotype

**Fig 3:**
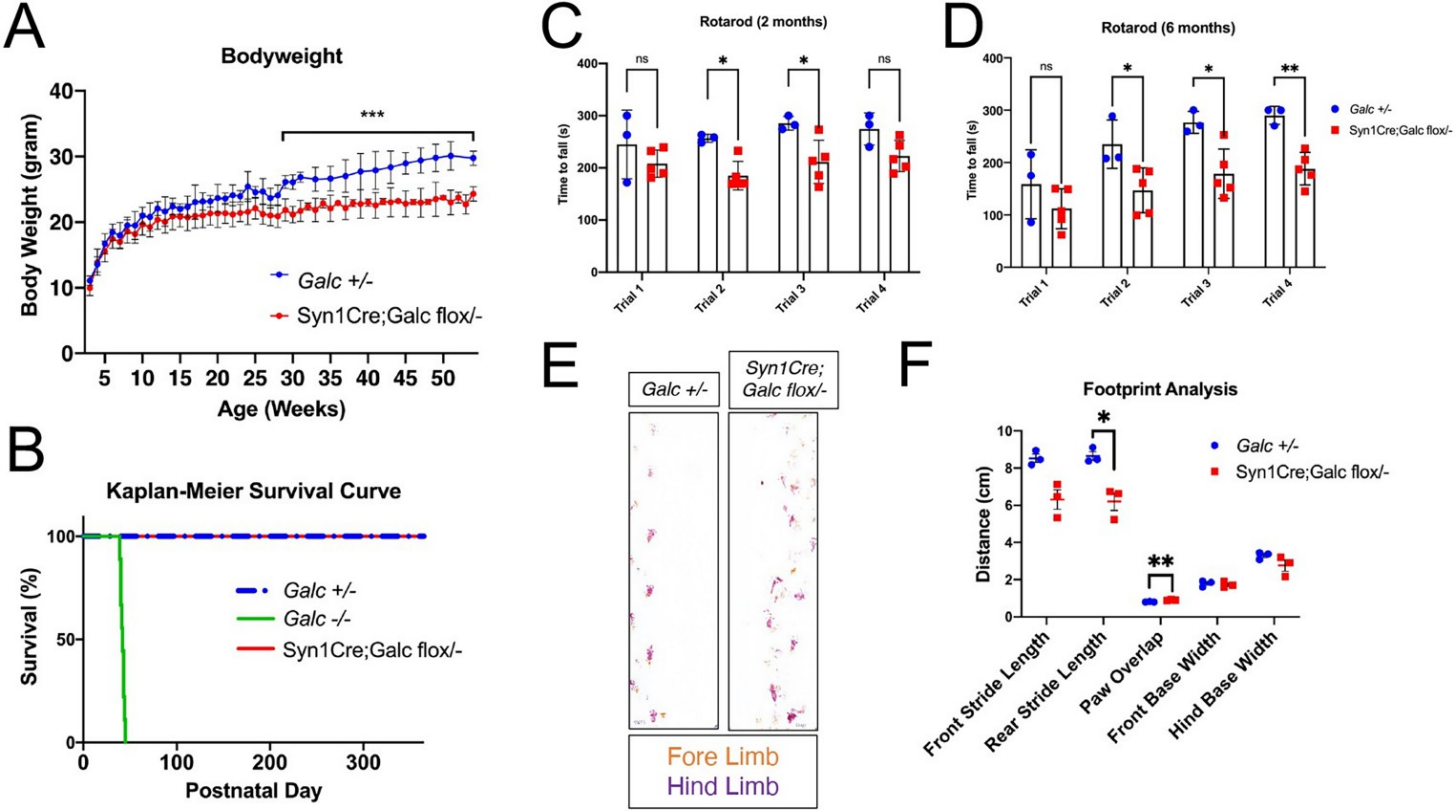
*Syn1Cre; Galc flox/−* mice have motor coordination defects with muscular wasting. (**A**) Body weight of *Syn1Cre; Galc flox/−* mice were dramatically reduced compared to control (*Galc +/−*). Female animals only were tested. *N* = 10 per genotype. (**B**) *Syn1Cre; Galc flox/−* mice survives more than 1 year, like control (*Galc +/−*). *N* = 15 each genotype. (**C, D**) Rotarod analysis of 2-month-old (**C**) and 6-month-old (**D**) animals shows that *Syn1Cre; Galc flox/−* mice had a poor performance, which was initially apparent at 2 months and became more pronounced at 6 months of age. Rotarod was performed with acceleration from 4 rpm to 40 rpm, for 300 seconds or to failure. *N* = 4 for *Syn1; Galc flox/−* and *N* = 5 for *Galc +/−*. Female animals only were tested. (**E**) Representative images of the footprint analysis of the 1.5-year-old *Syn1; Galc flox/−* and control (*Galc +/−*). Purple food-safe dye was used for the hind limbs, and orange food-safe dye was used for the forelimbs. Each mouse was given 3 trials of the test and the results from test 2 and 3 were pooled. *N* = 3. (**F**) Quantification of the footprint analysis shows impaired coordination in both paw placements, as measured in overlap, and a shorten rear limb stride length. Data are presented as mean values ± SEM; * *P* < 0.05, **P < 0.01. The underlying data for **A**–**D** and **F** can be found in S1 Data. GALC, galactosylceramidase.

The *Syn1Cre; Galc flox/−* mouse had significantly reduced body weight compared to control *Galc +/−*, but did not show any survival change (**Fig 3A and 3B**). Interestingly, as the *Syn1Cre; Galc flox/−* mice aged, we noticed ataxia and bradykinesia as well as a perceivable decrease in locomotion. Therefore, we performed the rotarod test to assess ambulation and coordination, at both 2 months and 6 months of age. We found a significant decrease in the ability of the *Syn1Cre; Galc flox/−* to perform the test compared to control *Galc +/−*, and older *Galc* mutant animals have more pronounced functional deficits (**Fig 3C and 3D**), similar to the global *Galc*-KO and *twitcher* (an authentic Krabbe mouse model) mice that display a degeneration of motor coordination [17]. We also performed a footprint analysis of the *Syn1Cre; Galc flox/−* mice to assess locomotive and coordination deficits (**Fig 3E and 3F**). The *Syn1Cre; Galc flox/−* mice had a significant decrease in their stride lengths and precision in their foot placement, suggesting either a functional motor deficit or a loss of proprioception. Because the hind limb stride length was more markedly decreased than the forelimb stride length, axonal signaling or axonal transport could play a role in this behavioral phenotype [28]. Defects in axonal transport have been seen in neurons lacking GALC, which aligns with the observation that the hindlimbs are more affected than the forelimbs, this is likely because of the longer axons projecting to the hindlimb [20]. The cerebellum plays a role in coordinating motor control and is also a site of pathology in Krabbe patients [29], but the sensorimotor cortex could also impact the performance of this test due to its role in proprioception [30]. Further behavioral testing could help to elucidate the brain regions that are most impacted by the loss of GALC.

### Neuron-specific *Galc* KO brains have morphological signs of neurodegeneration

**Fig 4:**
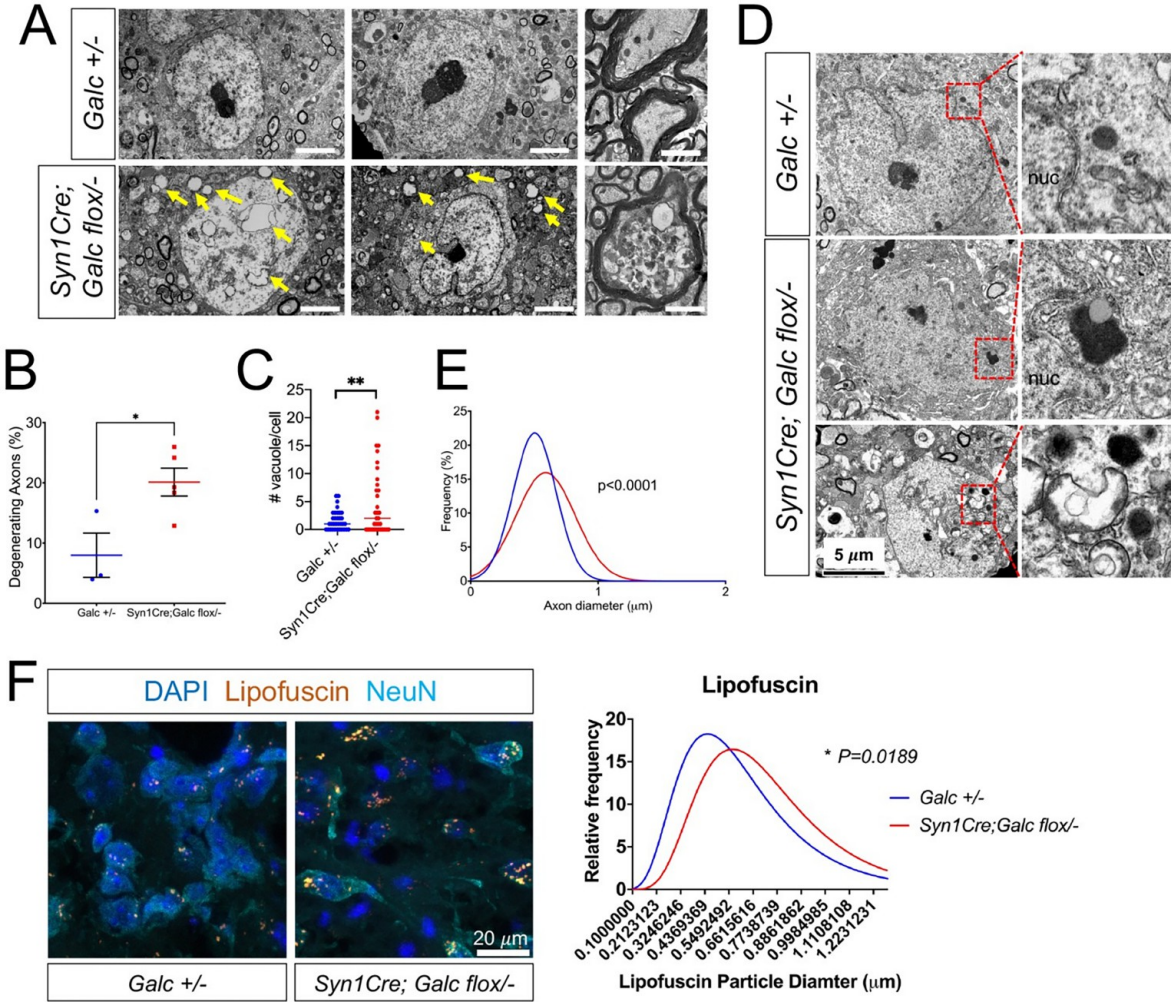
Neuron-specific *Galc* brains have morphological signs of neurodegeneration. (**A**) Electron microscopic analysis of the cervical spinal cords from *Syn1Cre; Galc flox/−* and control displayed an increased number of degenerating axons that contained many vacuoles (yellow arrows), compared to control (*Galc* +*/−*). Scale bar = 4 μm. (**B**) Quantification of degenerating axons showed that 20.1% of axons were degenerating in the mutant spinal cords, whereas only 7.8% of control axons had a degenerating phenotype. (**C**) *Galc-*deficient neuronal cells had a significant increase of vacuoles in the cytoplasm (yellow arrows in **A**) as compared to WT neurons. (**D**) Electron micrographs of mutant neurons and lysosomes (inset boxes in left panels and at higher magnification in right panels) show abnormal lysosomal morphology and inclusions in the neurons of *Syn1Cre; Galc flox/−*. nuc, nucleus. (**E**) The distribution of axon diameters was also significantly changed in the *Syn1Cre; Galc flox/−* mice, with a rightward shift, indicating larger axons. Data are presented as mean values ± SEM; * *P* < 0.05, **P < 0.01, ***P < 0.001, and ns, not significant. *N* = 3 for *Galc +/−* and 5 for *Syn1Cre; Galc flox/−*. (**F**) The signal of lipofuscin, a granule composed of lipid-containing residues of lysosomal digestion, was significantly increased in the neurons (NeuN) of 6-month-old *Syn1Cre; Galc flox/−* mice. The underlying data for **B**, **C**, **E**, and **F** can be found in S1 Data. GALC, galactosylceramidase; WT, wild-type.

Our previous study showed that brainstem region is most affected and has degenerating neurons by global *Galc* deletion [17]. Therefore, we analyzed thoroughly further the brainstem region of neuron-specific *Galc* mutant, to know if neuronal GALC affects neuronal phenotype autonomously. Interestingly, morphometric analysis of the brainstem from *Syn1Cre; Galc flox/−* exhibited a significant increase of degenerating and dying neurons (**Fig 4A and 4B**). Neuronal cell bodies of the mutants had an increase of vacuoles in the cytoplasm (**Fig 4C**), suggesting an increase of autophagic cell death. We also found abnormal lysosomal morphology in the *Syn1Cre; Galc flox/−* neurons, exhibiting enlarged and inclusion-filled lysosomes (**Fig 4D**). In addition, the median size of axonal diameter was slightly, but significantly, increased in the neurons from the *Syn1Cre; Galc flox/−* compared to control (*Galc +/−*) (**Fig 4E**). These enlarged axons may represent swollen axons that precede neurodegeneration [31]. Finally, lipofuscin, a granule composed of lipid-containing residues of lysosomal digestion, was highly accumulated in the neurons of 6-month-old *Syn1Cre; Galc flox/−* mice (**Fig 4F**), supporting a possible connection of Krabbe disease to lipofuscinosis and age-related neurodegenerative diseases [32].

**Fig 5:**
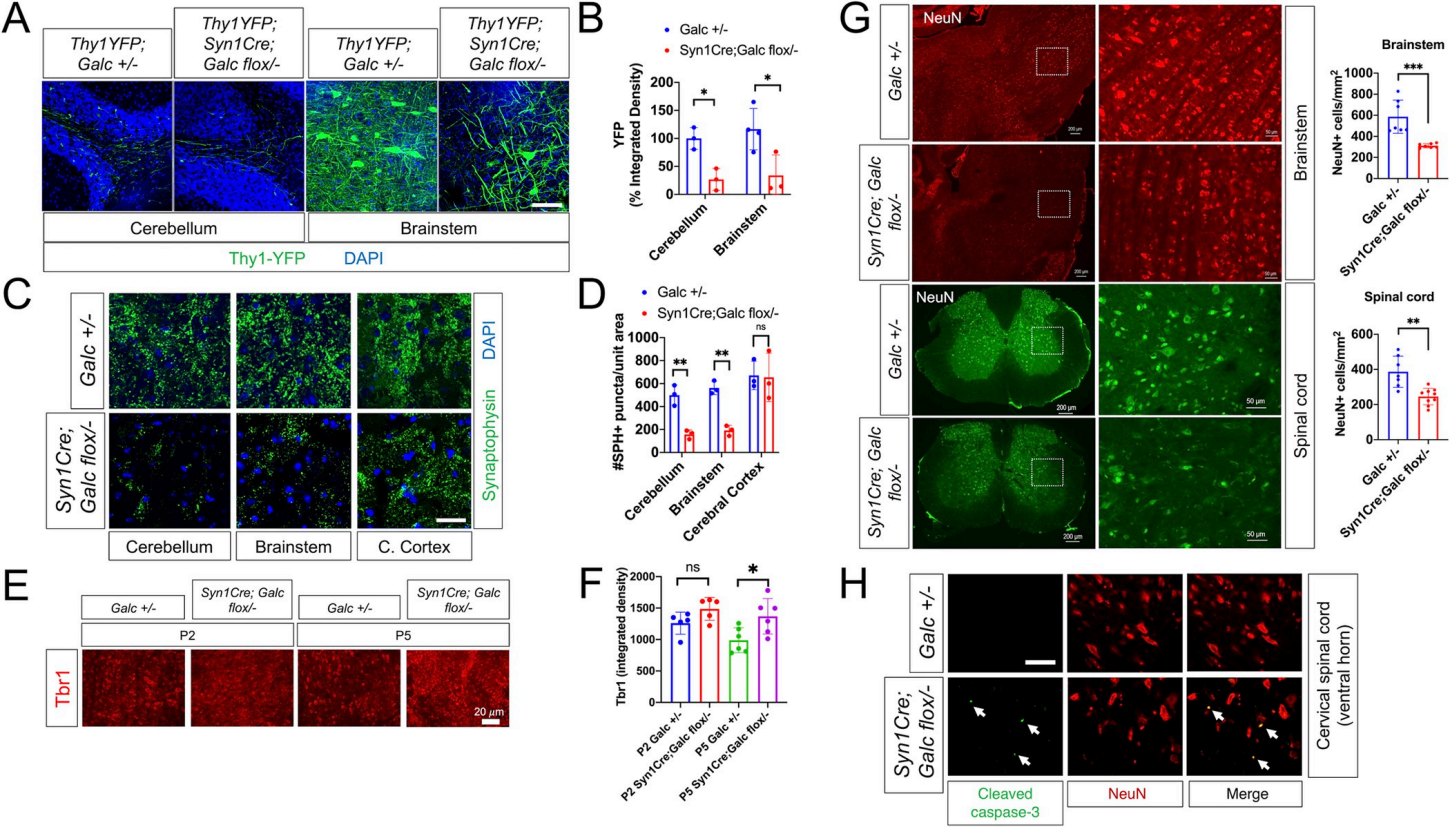
Neuronal complexity was greatly reduced by neuronal GALC depletion. (**A, B**) Double transgenic mice of 1-year-old *Syn1Cre; Galc flox/−;* Thy1.1-YFP shows that a decrease in the density of YFP neurons/axons in the brainstem and cerebellum, compared to control (*Galc +/−;* Thy1.1-YFP). Scale bar = 50 μm. (**C**) Puncta stained with SPH, a presynaptic marker, were counted by immunohistochemistry on brain sections of 6-month-old *Syn1Cre; Galc flox/−*. Scale bar = 50 μm. (**D**) Quantification of the puncta of SPH shows that their level was dramatically reduced in the cerebellum and brainstem, but not in the cerebral cortex of *Syn1Cre; Galc flox/−*, *N* = 3 per genotype. All data are presented as mean values +*/−* SEM. Two-way ANOVA with Tukey multiple comparison tests were used. *P < 0.05, **P < 0.01 and ***P < 0.001. ns, not significant. (**E**, **F**) Immature neuronal marker, Tbr1 expression is significantly higher in the brainstem of *Syn1Cre; Galc flox/−* mice, compared to *Galc +/−*. (**G**) NeuN-positive neuronal cell bodies were significantly reduced in both brainstem and spinal cord regions of 1-year-old mutant mice. (**H**) Immunostaining of cleaved caspase-3 (green) and NeuN (red) revealed that neuronal cell death is increased in the ventral horn of spinal cord (white arrows) of 6-month-old *Syn1Cre; Galc flox/−* compared to control. Scale bar = 50 μm. The underlying data for **B**, **D**, **F**, and **G** can be found in S1 Data. GALC, galactosylceramidase; SPH, Synaptophysin.

Neuronal swellings and varicosities occur prior to overt CNS demyelination in the *twitcher* mouse [16,33]. In addition, neural development is perturbed in *Galc*-KO brains, before evident microglial pathology starts. Sometimes, *Galc*-mutant axons had swellings, breaks, or transections, indicating severe axonal degeneration [17]. However, due to the ubiquitous nature of GALC, any previous studies were not able to discern whether these neuro-axonal pathology is neuron-autonomous or only secondary to other cell’s phenotype such as demyelination. To test if the abnormal neuro-axonal structures in Krabbe brain are due to the loss of neuron-autonomous GALC, we used Thy1.1-YFP reporter mice that express YFP sparsely in motor, sensory, and some central neurons [34]. Confocal analysis of the brains of Thy1.1-YFP crossed with *Syn1Cre; Galc flox/−* showed a significant decrease in the density of YFP axons in the brainstem and cerebellum of the 1-year-old mutant mice compared to control (**Fig 5A** and **5B**). Furthermore, a presynaptic marker, Synaptophysin (SPH), was much less present in the brain of *Syn1Cre; Galc flox/−* (**Fig 5C and 5D**), suggesting abnormal axonal structures in neuronal GALC-deficient brains.

Our previous study showed that temporal *Galc* deletion in perinatal neurons reduced neuronal maturation [17]. To ask if the neuroaxonal defect in *Syn1Cre; Galc flox/−* is also due to the delayed maturation, we assessed T-brain-1 (Tbr1) transcription factor expression in perinatal brains. Tbr1 is highly expressed in immature neurons during brain development and is gradually decreased as neurons mature [35]. Interestingly, the overall intensity of Tbr1-positive cells was also higher in the brainstem of *Syn1Cre; Galc flox/−* compared to control at P5, and there was a trend toward an increase at P2 (**Fig 5E and 5F**), suggesting that neuronal maturation delay is one of the factors that generate the phenotype in *Syn1Cre; Galc flox/−* mice. Neuronal cell loss was discernible in both brainstem and spinal cord of 1-year-old mutant mice, which was assessed by counting NeuN-positive neuronal cell bodies (**Fig 5G**). In line with the reduction of neuronal cell populations, immunostaining of a cell death marker, cleaved caspase-3, showed increased neuronal cell death in the spinal cord of *Syn1Cre; Galc flox/−* compared to control (**Fig 5H**). Taken together, these data indicate that the ablation of neuronal *Galc* results in neuronal cell degeneration and loss.

### Neuron-specific *Galc* deletion triggers neuroinflammation

**Fig 6:**
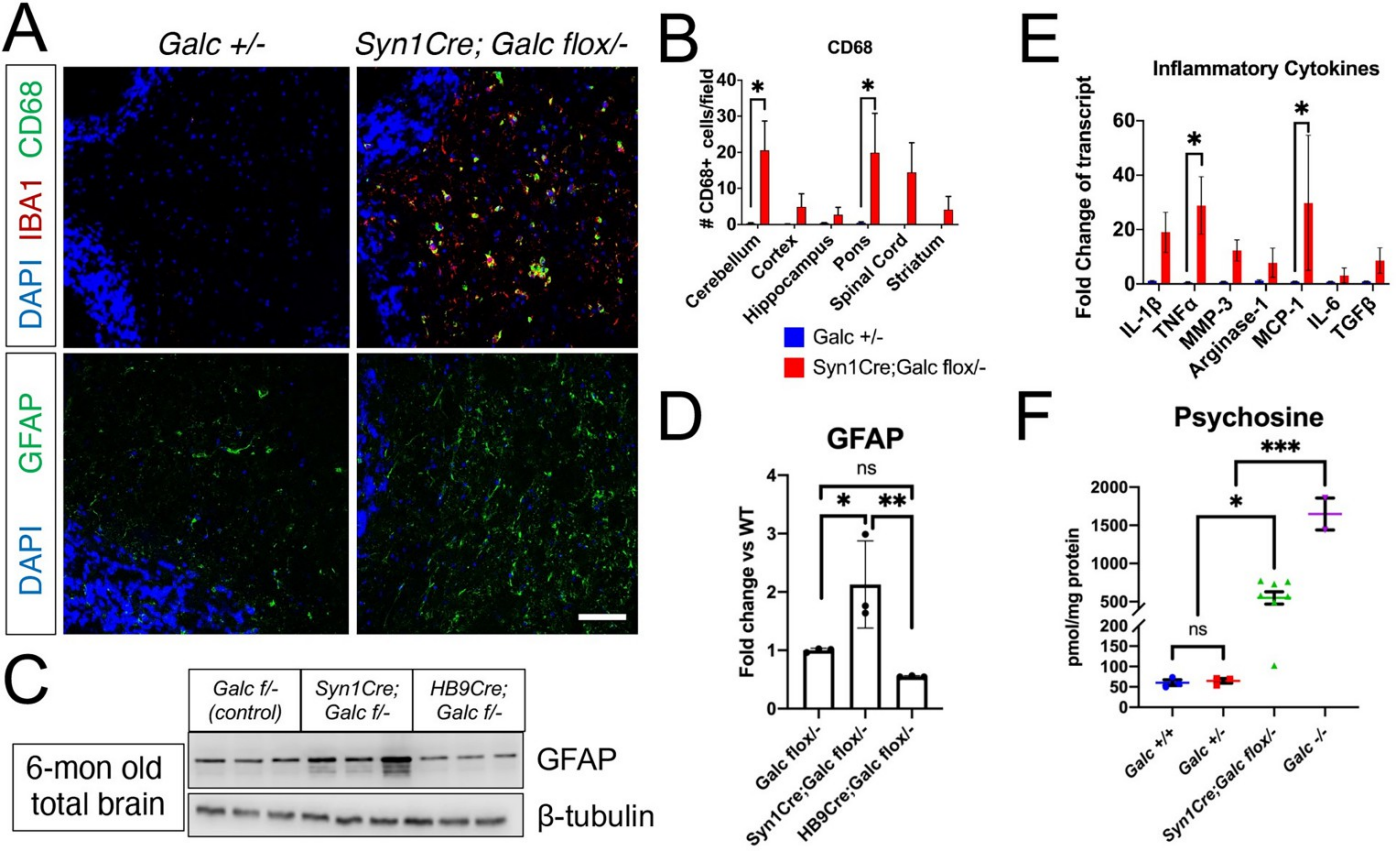
Neuron-specific *Galc* deleted mice have inflammatory gliosis with the accumulation of psychosine. (**A**) Immunohistochemistry on cryo-sections of cerebellar white matter shows activated microgliosis (Iba1 and CD68) and astrocytosis (GFAP), in the brain of *Syn1Cre; Galc flox/−*. Scale bar = 100 μm. DAPI is blue-colored. (**B**) Quantitative analyses shows that a phagocytic inflammatory marker CD68 was significantly higher in the cerebellum and pons of *Syn1Cre; Galc flox/−*, compared to control (*Galc* +*/−*) at 6 months old. *N* = 5. (**C**) Western blot analysis reveals a dramatic increase in the marker of astrocytosis (GFAP) in the brain of *Syn1Cre; Galc flox/−*. MN-specific *HB9Cre; Galc flox/−* has no increase of GFAP level in the brain. (**D**) Quantification of the protein band density shows an increase of GFAP in the *Syn1Cre; Galc flox/−*, but not in *HB9Cre; Galc flox/−*, compared to control (*Galc* +*/−*). (**E**) Quantitative reverse-transcriptase PCR analyses of inflammatory cytokines shows that there was a significant increase in TNFα and MCP-1 and moderate up-regulation of IL-1b, MMP-3, Arginase-1, IL-6 and TGFβ. *N* = 3 for *Galc +/−*, *N* = 4 for *Syn1Cre; Galc flox/−*, with 3 technical replicates. Data are presented as mean values ± SEM; * *P* < 0.05, **P < 0.01, ***P < 0.001, and ns, not significant. (**F**) Psychosine was measured in the cervical spinal cords of 6-month-old each substrain, except *Galc −/−* which were moribund P37-38. Psychosine level is significantly increased in the *Syn1Cre; Galc flox/−* compared to both *Galc +/+* and *Galc* +*/−*, although its level is only a third of the full KO *Galc −/−*. *N* = 7 for *Syn1Cre; Galc flox/−* and *N* = 3 for others. Data are presented as mean values ± SEM; *P < 0.05, **P < 0.01, ***P < 0.001, and ns, not significant. The underlying data for **B** and **D**–**F** can be found in S1 Data. GALC, galactosylceramidase; KO, knockout; IL, interleukin; MN, motor neuron; TGFβ, transforming growth factor beta; TNFα, tumor necrosis factor alpha.

A major hallmark of Krabbe disease is neuroinflammatory gliosis such as microgliosis and astrocytosis. To elucidate if inflammatory markers were up-regulated by neuronal GALC deficiency, we analyzed microgliosis (Iba1 and CD68) and astrocytosis (GFAP) markers by immunohistochemistry on brain sections of 6-month-old *Syn1Cre; Galc flox/−*. Interestingly, both CD68 and Iba1 were highly increased in most brain regions, especially in the hindbrain regions including the pons and cerebellum (**Fig 6A and 6B**). GFAP was also significantly up-regulated in the brain of 6-month-old *Syn1Cre; Galc flox/−* (**Fig 6A, 6C and 6D**). Furthermore, transcript levels of inflammatory cytokines were significantly increased in the brain of 6-month-old *Syn1Cre; Galc flox/−* (**Fig 6E**). These data suggest that neuron-specific *Galc* ablation dramatically induces neuroinflammation.

Neurological phenotypes of *Syn1Cre; Galc flox/−* mice shown in **Fig 3**, such as ataxia and bradykinesia as well as locomotive and motor coordination deficits, may imply a possibility of MN-autonomous pathogenesis. To ask if there is an involvement of MNs in the phenotype of *Syn1Cre; Galc flox/−*, MN–specific *Galc*-CKO mice were generated by crossing *Galc*-flox with *HB9Cre* transgenic mice (JAX#006600) [36]. However, MN–specific *HB9Cre; Galc flox/−* has no overt signs of inflammatory gliosis (**Fig 6C and 6D**). In theory, the lack of a strong phenotype may be due to a technical limitation in terms of recombination efficiency of *HB9Cre* mice. We think this explanation is unlikely due to the fact that *HB9Cre* mice are highly efficient (80% to 90%) at recombination in MNs (**S2 Fig**), which were tested in the tdTomato-reporter crossed with *HB9Cre* mice. They suggest that the phenotype of *Syn1Cre; Galc flox/−* may be mediated by non-MNs.

### Psychosine levels are elevated after neuron-specific *Galc* deletion

Psychosine accumulation is a major etiological driver of Krabbe disease, capable of inducing cell death of myelinating cells and neurons [37–39]. It has been suggested that psychosine can be generated by nonmyelinating cells including neurons in vitro [16,20], although the origin of the psychosine was not clear due to these neurons being derived from Krabbe tissues where there is a global loss of GALC. To elucidate the cause of the CNS phenotype in the *Syn1Cre; Galc flox/−*, we assessed psychosine level in the spinal cords of *Syn1Cre; Galc flox/−*, wild-type (WT; *Galc +/+*), heterozygotes (*Galc +/−*), and KO (*Galc −/−*) mice. High-performance liquid chromatography tandem mass spectrometry (LC–MS–MS) showed that, approximately 500 pmol of psychosine accumulated per mg of protein in the spinal cords of 6-month-old *Syn1Cre; Galc flox/−* mice. This was far higher than the psychosine concentration in control spinal cords (approximately 50 pmol/mg of protein) (**Fig 6F**), although it was 30% of the global *Galc-*KO level. There was no difference in the level of psychosine between *Galc +/+* and *Galc +/−*, indicating that haploinsufficiency is not sufficient to increase psychosine accumulation. These results suggest that *Galc*-deficient neurons produce psychosine in vivo, and this can directly contribute to the pathogenesis of Krabbe disease.

### Neuron-specific *Galc* deletion reduces CNS myelination marginally

**Fig 7:**
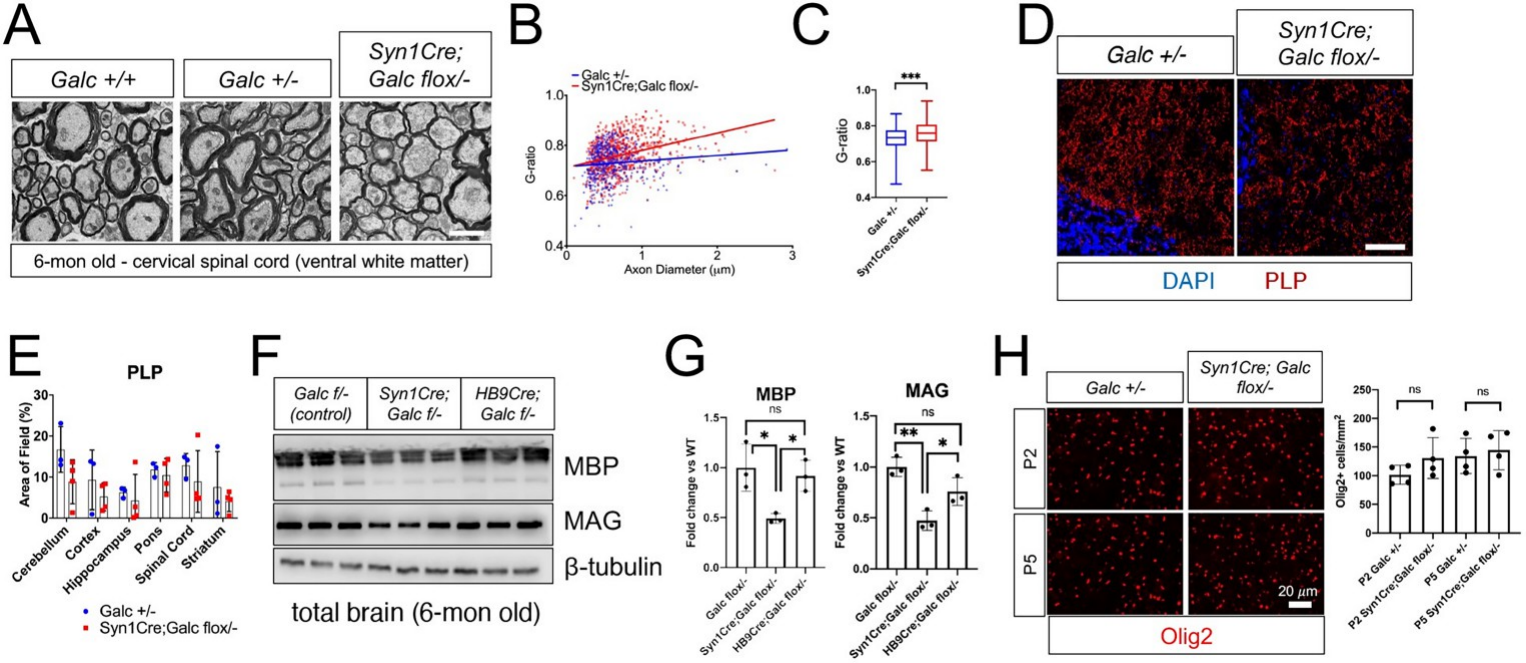
Myelin and myelin proteins are marginally reduced in the brain of *Syn1Cre; Galc flox/−*. Electron microscopy analysis of the cervical spinal cords (**A**) and their morphometry g-ratio quantifications (**B, C**) from 6-month-old *Syn1Cre; Galc flox/−* and controls (*Galc* +/+ and +*/−*). The myelin sheaths were significantly thinner in *Syn1Cre; Galc flox/−* versus controls; the mean g-ratio of *Galc* +*/−* was 0.72, whereas that of the mutant was 0.76. All data are presented as mean values ±SD. Welch *t* test was used; ***P < 0.001. The box bounds in the box plots for the g-ratio are 25th and 75th percentiles, the center lines are median value, and the whiskers are 0.05 and 0.95 percentiles. (**D**) Immunohistochemistry on brain sections shows that a decrease in the level of myelin protein PLP in the brains of 6-month-old *Syn1Cre; Galc flox/−*, compared to control (*Galc* +*/−*). Scale bar = 100 μm. (**E**) Quantification of PLP level, measured by averaged integrated signal intensity, reveals a trend of reduction in the mutant brain. (**F**) Western blot analysis revealed that myelin proteins such as MBP and MAG were reduced in the pan-neuron specific mutant *Syn1Cre; Galc flox/−*, but not in the MN mutant *HB9Cre; Galc flox/−*. (**G**) Quantification of the protein band density shows the dramatic reduction of MBP and MAG levels in the *Syn1Cre; Galc flox/−*, but not in *HB9Cre; Galc flox/−*, compared to control (*Galc* +*/−*). (**H**) Immunohistochemistry of Olig2-positive cells shows that there was no change in the number of OL lineage cells in the brainstem of *Syn1Cre; Galc flox/−*, compared to *Galc +/−*. *N* = 3. Data are presented as mean values ± SEM; *P < 0.05, **P < 0.01, ***P < 0.001, and ns, not significant. The underlying data for **B**, **C**, **E**, **G**, and **H** can be found in S1 Data. GALC, galactosylceramidase; MAG, and myelin-associated glycoprotein; MBP, myelin basic protein; MN, motor neuron; PLP, proteolipid protein.

To determine if the deficiency of neuronal GALC affects myelination, we also analyzed myelin thickness and the level of myelin proteins. Interestingly, scatter plots of the g-ratios of individual fibers in relation to respective axonal diameters and overall averaged myelin g-ratio assessment showed a thinner myelin in the mutants when compared to control littermates (**Fig 7A–7C**). Further analysis by immunohistochemistry (**Fig 7D and 7E**) and western blot analyses (**Fig 7F and 7G**) against myelin-specific proteolipid protein (PLP), myelin basic protein (MBP), and myelin-associated glycoprotein (MAG) showed a significant reduction of myelin proteins in the brain of *Syn1Cre; Galc flox/−* compared to control (*Galc* +*/−*), suggesting that neuronal GALC affects myelin maintenance in the CNS. Similar to the lack of phenotype seen in **Fig 6**, the MN-specific *Galc* deletion (*HB9Cre; Galc flox/−*) did not show any change of myelin proteins (**Fig 7F and 7G**).

We previously found that *Galc* expression is required for the proliferation of the OL population during brain development, although it was not affected by *Thy1Cre/ER*^*T2*^-mediated neuronal *Galc* ablation [17]. In this study, using *Syn1Cre* that causes constitutive deletion of neuronal *Galc*, we asked if the myelination change in the mutant is due to the effect of neuronal GALC on gliogenesis. In line with our previous result, analysis of Olig2-positive cell populations in the brainstem of *Syn1Cre; Galc flox/−* mice at perinatal P2 and P5 did not show any difference in the number of OL lineage cells (**Fig 7H**). This suggests that the myelin phenotype in *Syn1Cre; Galc flox/−* may not be due to gliogenesis, but instead toxicity from GALC-deficient neurons such as psychosine.

**Fig 8:**
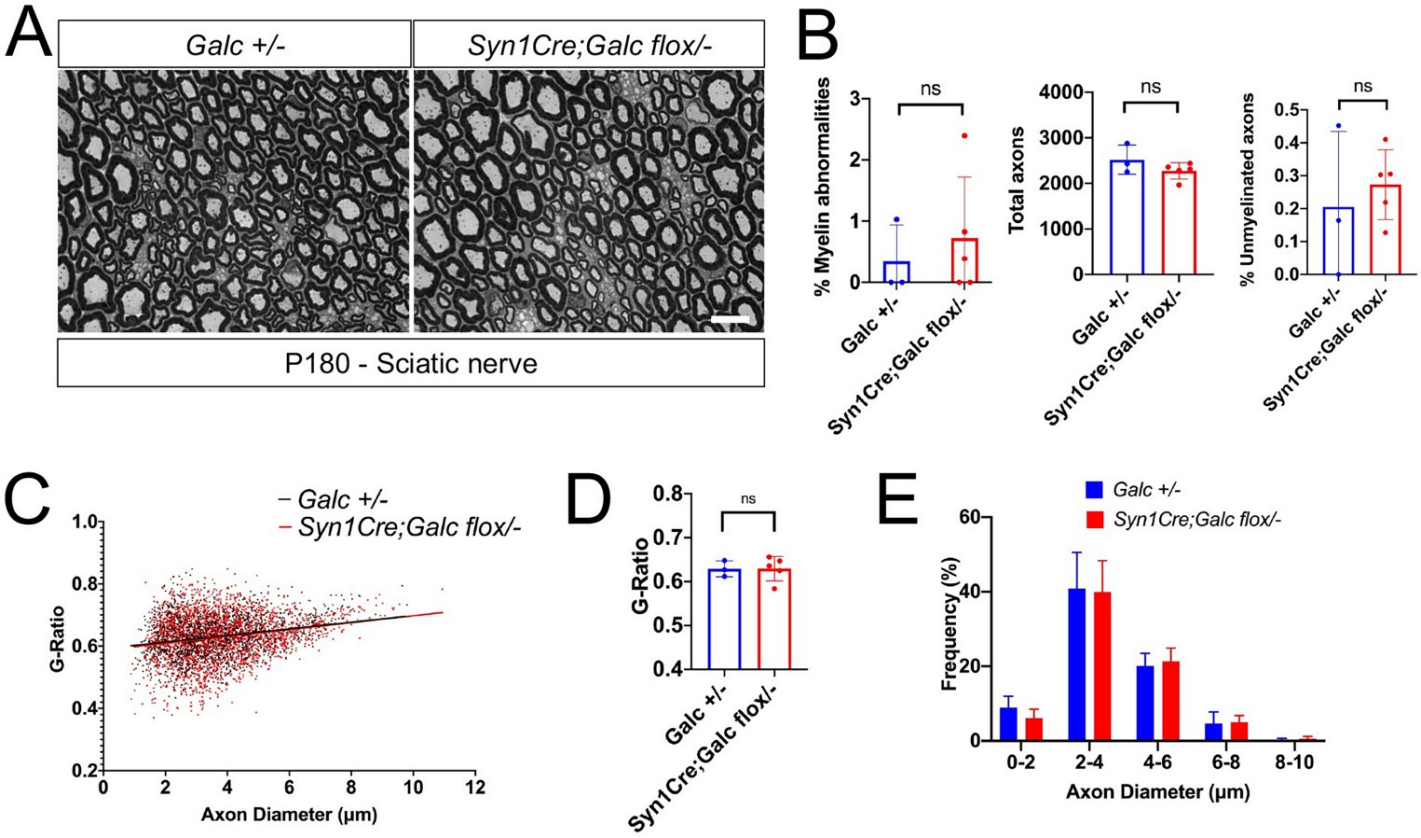
Sciatic nerve did not show any morphological change. (**A**) Representative semi-thin sections of P180 sciatic nerves from *Syn1Cre; Galc flox/−* (*N* = 5) and control (*N* = 3). Scale bar = 10 μm. (**B**) Myelin abnormalities were not significantly increased in the mutant. (**C–E**) Peripheral myelin thickness and distribution of axonal diameter were not changed in the *Syn1Cre; Galc flox/−*, compared to control (*Galc flox/−)*. *N* = 5. All data are presented as mean values +*/−* SEM. Two-way ANOVA with Tukey multiple comparison tests were used. *P < 0.05, **P < 0.01 and ***P < 0.001. ns, not significant. The underlying data for **B**–**E** can be found in S1 Data. GALC, galactosylceramidase.

As we and other groups reported, peripheral *Galc-KO* fibers are thinner and have myelin abnormalities, including myelin ovoids and sometimes onion bulbs, structures suggestive of ongoing demyelination and remyelination [20,40,41]. Therefore, to investigate if similar pathogenesis is triggered in the PNS of *Syn1Cre; Galc flox/−* mice, sciatic nerves of 6-month-old animals were analyzed. Syn1Cre was active in neuro-axons of the sciatic nerve (**S3A and S3B Fig**). At 6 months of age, *Syn1Cre; Galc flox/−* nerves did not show any signs of demyelination and other pathological hallmarks, such as myelin infolding, outfolding, decompaction, or onion bulbs compared with *Galc +/−* control (**Fig 8A and 8B**). The sciatic nerves of *Syn1Cre; Galc flox/−* did not have an increase in the number of unmyelinated axons, which was assessed by counting both myelinated and unmyelinated axons in entire nerves (**Figs 8B and S3C**). Also, as shown by g-ratio measurement, there was no change in the peripheral myelin thickness (**Fig 8C and 8D**) nor in the distribution of axonal diameter (**Fig 8E**), suggesting that the PNS is minimally or not affected by neuronal GALC.

## Discussion

We previously identified a critical perinatal period of vulnerability to GALC depletion in mice by inducing ubiquitous *Galc* ablation at various postnatal time points. During this vulnerable period, neuron-specific GALC influenced neuronal differentiation without affecting the OLs population. These data led to the discovery of a novel neuronal cell-autonomous role for GALC, which is sufficient to delay neuronal maturation. However, the induced neuron-specific *Galc* KO mouse did not show any pathological symptoms such as psychosine accumulation [17] or gliosis (**S4 Fig**). We suspected that this was because *Thy1Cre/ER*^*T2*^, while specific, was expressed in only a small subset of neurons, as many ER^T^-mediated Cre drivers are specific but lack robust and widespread Cre expression. Here, we decided to use the *Syn1Cre* mouse as a robust Cre driver among most neurons. In this study, we showed that GALC has a neuron-autonomous role, and its absence in neurons triggers neuro-axonal degeneration and subsequent inflammatory demyelination by affecting other brain cells.

Considering the more severe phenotype of the *Syn1Cre; Galc flox/−* mice compared to the previous *Thy1Cre/ER*^*T2*^*; Galc flox/−* model, the depletion of neuronal GALC is likely multifactorial wherein the constitutively active *Syn1Cre* is able to elicit further pathological consequences beyond the delayed neuronal maturation phenotype of the critical perinatal period observed in both models. (**Fig 5E and 5F**). It is possible that a specific type of CNS neuron is crucial for the phenotype of *Syn1Cre; Galc flox/−*. Since MN-specific *HB9Cre; Galc flox/−* mice had no abnormal phenotype (**Figs 6 and 7**), the critical neurons in the *Syn1Cre; Galc flox/−* pathology are likely non-MNs based in the CNS. Our prior study [17] revealed a subregion of the brainstem in which perinatal GALC is important. Therefore, our findings suggest that neuronal GALC has a primary role in neuronal homeostasis.

Disruption of neuronal homeostasis (**Fig 4**), caused by the ablation of neuronal GALC, influences myelin thickness and myelin protein levels (**Fig 7**). This happens, at least in part, independently of GALC absence in myelinating cells. No change in the number of OL lineage cells in the perinatal brains of *Syn1Cre; Galc flox/−* indicates that the myelin phenotype in *Syn1Cre; Galc flox/−* may not be due to loss of gliogenesis but presumably toxicity from GALC-deficient neurons such as psychosine (**Fig 7H**). A recent publication from Reiter and colleagues [42] demonstrated appreciable levels of psychosine in extracellular vesicles of the *twitcher* mouse, providing proof of concept to the idea that psychosine of neuronal origin may influence the health of neighboring cells. Alternatively, in line with recent studies showing that electrically active neurons can signal nearby OPCs to initiate proliferation and/or differentiation to mature OLs in response to environmental cues [43], it is possible that deletion of *Galc* in neurons alters neuronal activity and its effect on myelination. Moreover, it is possible that the survival of OLs may be dependent on the presence of healthy axons as shown by the death of approximately 50% of OLs in the optic nerve due to loss of axonal substrate [44]. Furthermore, the signals from axons that promote myelination must also be considered. Neuronal signals, such as neuregulins, Notch-1, neurotrophins, and polysialylated-neural cell adhesion molecules, from the axonal membrane regulate myelination, integrating signals from multiple axons, with different caliber and electrical activity (reviewed in [45]). In addition, neurons and glia interact via extracellular vesicles called exosomes that carry a variety of metabolites, proteins, lipids, mRNAs, and miRNAs [46]. Since these secretory organelles share the common lysosomal pathway, it is conceivable that dysfunctional lysosomal secretory pathways in neurons caused by *Galc* deletion may trigger aberrant communication between neurons and OLs, eventually affecting myelination.

We are the first to show that psychosine is accumulated in vivo in the *Galc*-deleted neurons (**Fig 6F**) and is accompanied by axonal degeneration and mild myelin loss, proving that neurons could be one of the primary cell types targeted by the pathology independently from other cell type contributions. The psychosine accumulation was not seen in our previous study using *Thy1Cre/ER*^*T2*^-mediated *Galc* ablation [17]. This discrepancy might be due to the fact that *Thy1Cre/ER*^*T2*^ only mediates temporal GALC depletion with tamoxifen whereas *Syn1Cre* is constitutively active. It may be the case that a majority of neuronal psychosine is generated early in neurogenesis when neurite extension and membrane synthesis is at its height. If that is the case, then the *Thy1Cre/ER*^*T2*^ model would miss the window of peak neuronal psychosine formation whereas *Syn1Cre* would permit psychosine generation prenatally. Furthermore, considering the transient persistence of tamoxifen, while many neurons would be subject to recombination, the effects of this could be diluted out by neurons differentiated past this window. The accumulation of psychosine is proposed to be responsible for significant pathology found in Krabbe disease. Psychosine can intercalate into membrane microdomains in the brain of Krabbe patients, disrupting the lipid raft structure [5], which is highly enriched in signal-transducing proteins and thus affects multiple signaling pathways critical to neuronal development, function, and axonal guidance [47, 48]. Specifically, psychosine abolishes the protein kinase C signal pathway [5], which is critical for synaptic plasticity as well as myelination in OLs [49,50]. Psychosine also impairs the axonal transport and synaptic structures by dephosphorylating neurofilaments [51,52]. Since synaptic terminals are most exposed to astrocytic and microglial processes, accumulation of psychosine at the synapse would trigger a cascade of responses leading to exacerbated neuroinflammation [53,54] and neurodegeneration as seen in the neuron-specific *Galc*-CKO brain (**Figs 4** and **6**). This possibility is also supported by a recent finding that GALC-deficient immature neurons are more vulnerable to accumulated psychosine, less responsive to external signals, and eventually eliminated [17,55]. Last, it is possible that psychosine may alkalize the lysosomal pH in GALC-deficient neurons, as shown in *Galc*-KO OPCs [8]. It is likely that the lysosomal reacidification that rescued OPC proliferation would be beneficial to neuronal health as well. However, despite a significantly reduced body weight along with the neurological phenotype, neuron-specific *Galc*-CKO mice did not show a reduced life span as with the global *Galc-*KO [17] (**Fig 3**), indicating that the nonneuronal autonomous process is critical to make a full Krabbe disease phenotype.

Psychosine is known to be formed in at least 2 different ways: by ceramide galactosyltransferase (UGT8) via galactosylation of sphingosine [14], or by acid-ceramidase with deacylation of GalCer [2]. While it is true that UGT8 is highly enriched in OLs and Schwann cells, there is evidence via in situ hybridization that the *UGT8* transcript exists at low levels in particular subsets of neurons including in the deep cerebellar nuclei, lateral cerebellar nuclei, and lateral vestibular nuclei [56]. These neurons should therefore have the capability for de novo (and autonomous) synthesis of psychosine from galactose and sphingosine. Alternatively, it is possible that psychosine can be generated from neuronal GalCer. Our preliminary colocalization study of GalCer (O1 antibody) with Npas4 (neuronal marker) in the murine brain shows the presence of neuronal GalCer (**S5 Fig**), suggesting GalCer is a component of neuronal cell membranes. A number of studies highlight the importance of GalCer in neurons. In juvenile neuronal ceroid lipofuscinosis, which is caused by mutations in the *CLN3* gene, neuronal GalCer expression and transport is important and thus a defect in the anterograde transport of GalCer from the trans-Golgi to lipid rafts of plasma membrane affects the proper composition, structure, and function of plasma membranes, which, in turn, leads to deregulation of ceramide levels with an end effect of increased neuronal apoptosis [57]. Since acid ceramidase is expressed ubiquitously, it is conceivable that psychosine can be generated from the deacylation of neuronal GalCer [2]. Last, recent evidence indicating the presence of OL-derived exosomes that traverse the periaxonal space to be internalized by axons could provide a source of extra-neuronal GalCer [58]. These exosomes have been shown to deliver the NAD-dependent deacetylase SIRT2 to promote local ATP production. The import of these transcellular exosomes can provide a route for GalCer to be ushered into neurons, which could be exacerbated in the case of axonal dysfunction.

Although *Syn1Cre; Galc flox/−* mice had neuronal pathology with central myelination defects, they did not show overt signs of demyelination or other pathological hallmarks in the PNS. These mice had largely unchanged nerve morphology including normal axonal numbers and density, as well as normal myelin thickness and appearance. This is surprising, as *Syn1Cre* was efficiently expressed in the PNS (**Figs 8 and** S3). Widespread demyelination and edema are prevalent in the peripheral nerves of Krabbe models and patients [20,40,41]. Of note, Teixeira and colleagues [20] showed that the sciatic nerves of *twitcher* mice had fewer axonal numbers before demyelination and endoneurial edema was evident, which seems not caused by a developmental impairment since the axonal density was not changed at birth. Our prior work [40] also showed that axonal reduction of global *Galc* KO nerves was quantified to approximately 10% in the entire nerve cross-section. The discrepancy between the current study and other previous studies may imply that the reduced myelinated axons in the *twitcher* model is caused not only by neuron-autonomous GALC but also by other cells’ effects and interplay including myelinating cells.

Lipofuscin pigment granules, which reflect lipid-containing residues of lysosomal digestion, are found in the cytoplasm of neurons in patients with neurodegenerative diseases like Alzheimer, Parkinson, and some lysosomal storage diseases [32,59]. We found increased levels of lipofuscin in the brains of *Syn1Cre; Galc flox/−* mice (**Fig 4F**), suggesting an underlying similarity between this mouse model and other neurodegenerative diseases. In line with this, the *GALC* gene was found to be a disease risk locus for Parkinson disease, along with other lysosomal genes such as glucocerebrosidase (*GBA*) [60]. Therefore, lysosomal dysfunction is likely involved in the underlying pathogenesis of Parkinson disease. Furthermore, the role of lysosomal biology in the degradation of protein aggregates has recently emerged in the risk of developing Parkinson disease. The most common Parkinson disease–related mutation is *GBA*, the enzyme defunct in Gaucher disease, with many patients and carriers of Gaucher disease develop Parkinsonian symptoms in later life (reviewed in [61]). It is therefore reasonable to suspect that perturbed lysosomal function of dopaminergic neurons may be related to the pathology and symptoms of *Syn1Cre; Galc flox/−* mice. These data would also correlate with a number of recent studies that show GALC deficiency is associated with other pathologic signs of Parkinson disease [62–64]. The aggregated forms of alpha-synuclein and ubiquitin, which are involved in the formation of Lewy bodies in Parkinson disease, are accumulated in the brains of both Krabbe patients and the *twitcher* mouse model. Therefore, our findings of the increased lipofuscin and lysosomal inclusions in the brains of *Syn1Cre; Galc flox/−* mice suggest a potential role of neuronal GALC in age-related neurodegenerative diseases.

Using Thy1.1-YFP reporter mice that can visualize neuro-axonal structures [34], we found a dramatic reduction of YFP signals in the neuron-specific *Galc*-CKO brain (**Fig 5A and 5B**), similar to the phenotype seen in global *Galc*-KO animal models [17]. The parallel finding of decreased presynaptic SPH in the mutant brain (**Fig 5C and 5D**) suggests diminished synaptic structures due to *Galc* deficiency. In fact, it has been reported that neuronal lysosomes regulate synaptic structures and activity [65,66]. For example, they dynamically modulate dendritic spine numbers in an activity-dependent manner [67,68]. Moreover, neuronal lysosomes undergo regulated exocytosis to aid in the membrane expansion during dendrite formation, ultimately aiding in the plasticity of dendritic spines [69]. Neuronal lysosomes also regulate the fate of the α-amino-3-hydroxy-5-methyl-4-isoxazolepropionic acid (AMPA) receptors to control the excitatory synaptic signal strength [70]. Finally, postmitotic neurons require constant membrane remodeling, in which lysosomes are imperative to release and uptake synaptic vesicles [71,72]. Therefore, it is highly likely that GALC-deficient lysosomes may be dysfunctional, which results in the aberrant synapse and dendritic spines leading to neuronal degeneration and abnormalities.

A prominent feature of Krabbe disease is neuroinflammation that includes widespread gliosis and exponential increase of proinflammatory chemokines [73]. Our neuronal *Galc* deletion also induces inflammatory gliosis with increased proinflammatory cytokines in the brain (**Fig 6**), akin to the phenotype seen in genuine Krabbe animal models and patients [17,40]. Although immune surveillance and protection via the propagation of inflammatory responses are the main functions, microglia also modulate synaptic pruning and learning, demonstrating their benefit and necessity to normal brain function [74]. However, there is a downside as these cells can increase the permeability of the blood–brain barrier, and thus, their overrecruitment can be destructive, causing a loss of myelin and neuronal death [75,76]. Our previous finding in which inflammation is elicited by myelin debris in *Galc*-deficient macrophages [40] allows us to speculate that the inflammation seen in the neuronal *Galc*-CKO model may be mediated by degenerated neurons and axons.

In summary, our findings highlight that GALC-deficient neurons can generate psychosine in vivo, which may, in turn, induce neuronal death, inflammation, and myelin loss. We confirmed that OLs are not the sole cell type responsible for initiating Krabbe pathogenesis, as *Galc*-ablated neurons contribute in a cell-autonomous manner to disease progression and are sufficient to cause some component of the disease. This may indicate the presence of specific cellular mechanisms of GALC function in neurons that need to be corrected to cure the disease. Furthermore, Parkinson disease–like pathology in the neuron-specific *Galc* KO such as lipofuscin accumulation may provide evidence of the connection between GALC dysfunction and the pathogenic mechanism of other neurodegenerative diseases. Further studies will elaborate on the specific molecular mechanisms in which neuronal *Galc* deletion triggers cellular and lysosomal pathogenesis in Krabbe disease and if similar processes occur in other lysosomal storage diseases.

## Materials and methods

### Animals

Experiments were conducted according to the protocols approved by the Institutional Animal Care and Use Committee of University at Buffalo—SUNY and Roswell Park Cancer Institute (protocol approval nos. UB1188M, UB1254M, and PROTO202000063). Mice were housed under specific pathogen-free conditions at 70°F, 50% room humidity, 12-hour light/12-hour dark cycle and received ad libitum access to water and food. All animals were maintained on the congenic background of C57BL/6N. Breeder C57BL/6N mice were purchased from Charles River (Wilmington, Massachusetts, United States of America). *Galc-flox* mouse line was generated as described in [17]. Syn1Cre (JAX#003966), HB9Cre (JAX#006600), Thy1.1-YFP (JAX#003782), TRAP line or EGFP-L10a (JAX#024750), and tdTomato (JAX#007905) mice were purchased from The Jackson Laboratory (Bar Harbor, Maine, USA). Since Syn1Cre transgene expression in the testes of male mice can produce germline recombination in the progeny [77], we used only female Syn1Cre mice for breeding. To maximize the GALC depletion effect, the haplodeficient *Galc* heterozygote: *Syn1Cre; Galc flox/−* was used, with controls *Galc +/−* or *flox/−*. *Galc* heterozygotes (*+/−*) did not show any difference to *Galc +/+* in respect of the myelin morphology and level of myelin proteins, as shown in **Figs 7A** and **S6A**. Also, the expression of myelin proteins such as MAG and MBP was not different between both haplodeficient *Galc +/−* and *Galc flox/−*, as shown in **S6B Fig**, suggesting the unrecombined floxed allele itself does not affect myelin phenotype.

### Survival and body weight measurements

Animals were weighed biweekly to record their mass using a plastic beaker and scale, in this study, only the weights of females were graphed to prevent any gender-related differences. These weight measurements also enabled survival tracking, in which littermates or closely born litters were grouped into cohorts by genotype.

### Rotarod analysis

Motor coordination and balance were tested using an accelerating rotarod (Ugo Basile, Italy). Only female mice were used to prevent gender differences from affecting the results. All mice were tested in 2 sessions of 4 trials each per day (6 hours rest between the 2 daily sessions) for 2 consecutive days. Analyses measuring P60 animals occurred at P59 and P60, those measuring 6-month-old animals occurred at P179 to P180. Only naïve mice were used for Rotarod tests (no training occurred prior to trial 1). Rotarod conditions were set to an acceleration of 5 rotations per minute, starting at a minimum velocity of 4 rotations per minute and accelerating to a maximum velocity of 40 rotations per minute. Each trial consisted of one acclimating run that was not scored. The next 3 runs were recorded and averaged. Each run was stopped when the mouse fell or passed completely underneath the rod (180 degrees of rotation).

### Footprint analysis

One-year-old animals of both genders were used for the footprint analysis as described in [28]. Briefly, mice were made to traverse a straight, well-lit runway (30-cm long, 10-cm wide) to receive food in a darkened box at the far end. The bottom of the runway was lined with white paper and the fore- and hind paws were inked with food-safe paints of orange and purple, respectively. A minimum of 3 nonstopping passes were required. The distance between 3 sets of R/L forelimb/hindlimb strides were measured along with the distance between fore- and hindlimbs overlap to measure the accuracy of foot placement.

### Tissue and immunohistochemistry

Mice at defined ages were anesthetized, humanely killed, and then perfused with ice-cold PBS followed by 4% PFA. If the tissue was for protein or RNA analysis, only PBS was perfused, and the tissue was quickly frozen in liquid nitrogen and stored at −80°C for later analysis. Brains and spinal cords were dissected, postfixed in 4% PFA for 48 hours, dehydrated in 30% sucrose at 4°C, embedded in OCT (Leica, Wetzlar, Germany), and processed as cryosections with a thickness of 25 μm. For immunohistochemistry, cryosections were rehydrated at room temperature for 15 minutes in PBS, then permeabilized and blocked in 0.1% Triton X-100, 3% bovine serum albumin, and 2% normal goat serum for 1 hour at room temperature. For GALC immunostaining, the blocking buffer also contained 0.05% saponin. The primary antibodies were then incubated overnight at 4°C. After washing (3× for 10 minutes) with PBS, sections were incubated with fluorophore-conjugated secondary IgGs (Jackson Laboratories, Maine, USA). After washing with PBS, coverslips were mounted with Vectashield (Vector Laboratories, California, USA) mounting medium and DAPI. Primary antibodies used were GALC [78], NeuN (EMD Millipore, Missouri, USA), Olig2 (Proteintech, Illinois, USA), CC1 (EMD Millipore), GFAP (Sigma-Aldrich, Massachusetts, USA), Iba1 (Wako, Japan), CD68 (Bio-Rad, California, USA), PLP (hybridoma AA3), SPH (Sigma-Aldrich), Tuj1 (BioLegend, California, USA), ChAT (Abcam, Cambridge, United Kingdom), cleaved caspase-3 (Cell Signaling Technology, Massachusetts, USA), CNPase (Abcam), Tbr1 (Abcam), GalCer/O1 (Millipore-Sigma, Massachusetts, USA), Npas4 (MyBioSource, California, USA), and MBP (EMD Millipore). Lipofuscin staining was done according to [79]. Briefly, cryosections were treated with lithium borohydride (LiBH4) to block background autofluorescence and then costained with NeuN to identify lipofuscin puncta within neurons. Confocal microscopic images were segmented to only include lipofuscin particles within the neuronal boundaries. The minimum ferret diameter of the particles was measured and analyzed to generate distributions of the sizes of lipofuscin particles to measure lipofuscin accumulation. All images were acquired and analysis was performed blinded to genotypes. Image quantification was performed using ImageJ (NIH). Maximum intensity images were segmented with *ilastik* (v1.4b3) [80]. The segmented images were processed with ImageJ (NIH) to quantify the intensity of the GALC staining per cell.

### TRAP

Total RNAs were purified from ribosomes, which were pulled down from snap-frozen total brains according to [81]. Briefly, brains were removed and snap-frozen in liquid nitrogen and stored at −80°C until all samples were collected. Total brains were homogenized in ice-cold polysome extraction buffer. Monoclonal anti-GFP antibodies (Monoclonal Antibody Core Facility, Memorial Sloan-Kettering Cancer Center, New York, New York, USA) were coupled to Dynabeads (Thermo Scientific, Massachusetts, USA) and added to brain supernatant. Beads and extract were incubated at 4°C, with agitation, for 30 minutes. The beads were then washed through a large volume of wash buffer, resuspended in lysis buffer, and RNA isolated; contaminating DNA was removed by DNaseI digestion (QIAGEN RNeasy Mini Kit, Hilden, Germany). The integrity and yield of the final RNA preparation were determined by Nanodrop 2000 (Thermo Scientific) measurement.

### RT-qPCRs

RNA was isolated using TRIzol reagent following the manufactures instructions (Thermo Scientific). RNA was then analyzed for purity on a NanoDrop 2000 spectrophotometer (Thermo Scientific). cDNA was synthesized from 500 ng of RNA using the Superscript III kit (Thermo Scientific) according to the manufacturer’s instructions. qPCR was performed using the SYBR Green PCR Master Mix (Applied Biosystems) with β-actin as a reference. All samples were analyzed with at least 3 technical replicates, and the relative expression was calculated using the ΔΔC_t_ method as described in [82].

### Cell counts and z-stacks

The cell type composition was assessed in matched sagittal brain sections (N > 3 mice) selected within the region containing each cell-specific marker (NeuN for neurons; GFAP for astroglia; Olig2 and CC1 for oligodendroglia; Iba1 for microglia) and nuclear counterstaining (DAPI) in immunofluorescence followed by Leica SP5 confocal microscopic analysis. z-stacks were recorded utilizing sequential confocal images that were collected at 2 μm intervals covering 30 μm depth. Numbers for each cell type were acquired by manually counting cells.

### Morphological analysis

Mice were anesthetized with 250 mg/kg body weight avertin (Sigma-Aldrich) and then perfused with PBS and 2.5% gluta-aldehyde in phosphate-buffer, and stored at 4°C until processing. After being postfixed, spinal cords and pons were dissected, incubated in 1% osmium tetroxide, dehydrated using sequential incubation in ethanol of increasing concentration, and embedded in Epon resin using propylene oxide as a transition solvent. Semithin sections were cut with 1 μm thickness and stained with 2% toluidine blue. Ultrathin sections of 80 to 85 nm thickness were stained with uranyl acetate and lead citrate to be examined by a Tecnai electron microscope. For g-ratio analyses of myelin in pons, ultrathin EM images were captured at x2900 and manually measured using FIJI (NIH). For g-ratio analyses of sciatic nerves, images were acquired from semithin sections using the ×100 objective of a Leica DM6000 microscope. Axon and fiber diameters were calculated using a semi-automated protocol in the Leica QWin software (Leica Microsystem, Wetzlar, Germany).

### Western blot analyses

After homogenizing whole brains in pre-chilled RIPA (Radio-Immuno-Precipitation Assay) lysis buffer containing protease inhibitors (Roche, Basel, Switzerland) and PMSF, total protein extracts were separated by SDS-PAGE, transferred to PVDF membrane (EMD Millipore), and blocked with 5% Skim milk or BSA in TBS-Tween20. Primary antibodies used were β-tubulin (Novus Biologicals, Colorado, USA), GFAP (Sigma-Aldrich), CNPase (Cell Signaling Technology), MAG (Zymed, California, USA), and MBP (EMD Millipore). Specific protein bands were quantified utilizing FIJI (NIH) and Image Studio (LI-COR Biosciences, Nebraska, USA), and the values (in pixels) obtained were normalized on those of the corresponding β-tubulin bands. Normalized values were then expressed as the percentage of values obtained from matched bands of control tissues.

### GALC enzyme assay

GALC activity was determined by the previously described method [83]. Briefly, snap-frozen whole brains were homogenized in 10 mM sodium phosphate buffer, pH 6.0, with 0.1% (v/v) Nonidet NP-40 by using a Dounce homogenizer. 1 μg of total brain lysates were mixed with 4-methylumbelliferone-β-galactopyranoside (final 1.5 mM) resuspended in 0.1/0.2 M citrate/phosphate buffer, pH 4.0, and AgNO_3_ (final 55 μM) at 37°C for 1 hour. The enzymatic reactions were stopped by adding 0.2 M glycine/NaOH, pH 10.6. Fluorescence of liberated 4-ethylumbelliferone was measured on a spectrofluorometer (λ_ex_ 360 nm; λ_em_ 446 nm). The GALC activity can be affected by the conditions of preparation and storage of tissue samples, giving a slight variance between studies. Therefore, all control tissues and specimens were handled and stored identically, so the relative changes of GALC activity are consistent.

### Measurement of psychosine

Psychosine level in tissues were analyzed as described in [40, 84]. Briefly, cervical spinal cords were homogenized in PBS. A fraction of PBS-homogenate was refrozen and shipped for analysis by Michael Gelb’s lab at the University of Washington. The other fraction of PBS-homogenate was mixed with 10X RIPA lysis buffer (Cell Signaling Pathway) to make 1X RIPA buffer. Samples were then sonicated and analyzed for protein quantification. For psychosine analysis, 250 μL of 1 nM d_5_-psychosine (Avanti Polar Lipids, Alabama, USA) in methanol was added to 5 μL of tissue/PBS homogenate. Psychosine was extracted at 37°C for 2 hours with orbital shaking and subsequent centrifugation. The supernatant was loaded onto a methanol-preconditioned Oasis MCX column (1 cc, 30 mg, Waters, #186000252, Massachusetts, USA). After sample loading, the cartridge was washed with 1 mL of water with 2% formic acid, 1 mL of methanol, and then 1 mL of 80:20 methanol:water (v:v) with 5% NH_4_OH. The column was washed with 0.8 mL of methanol with 5% NH_4_OH, which was collected and solvent evaporated using a SpeedVac vacuum concentrator. The residue was reconstituted with 100 μL mobile phase B prior to UPLC-MS/MS analysis. An ACQUITY UPLC I-Class system from Waters was used for the separation of glucosyl- and galactosyl-sphingosine (psychosine). The UPLC system was coupled to a Xevo TQ-S (Waters) tandem mass spectrometer, which was operated in the multiple reaction monitoring (MRM) mode.

### Primary mixed MN cultures

For primary mixed MN cultures, the ventral spinal cord was dissected from 12.5- to 13.5-day-old mouse embryos, based on the protocol outlined in [85]. Briefly, pregnant females were humanely killed and the uterus removed. Embryos were removed from the amniotic sacs and transferred to pre-chilled Hanks’ Balanced Salt Solution (HBSS, Ca^2+^ and Mg^2+^ free) containing penicillin and streptomycin. After removal of the head, the neural tube was separated from the rest of the embryo under a light dissection microscope. Ventral horns were pooled and digested in trypsin. The tissue was then triturated 4 times in DNase I in Leibovitz’s L15 medium and centrifuged with a 4% BSA cushion in Leibovitz’s L15 medium in order to pellet cells. The resultant cell pellet was re-suspended in supplemented neurobasal medium containing 2% B27 supplement, 0.5 mM GlutaMAX, 25 μM 2-mercaptoethanol, 2% horse serum, 0.1 ng/ml murine glial cell line-derived neurotrophic factor (GDNF, Peprotech), 0.5 ng/ml human or rat ciliary neurotrophic factor (CNTF, Peprotech), 0.1 ng/ml human brain-derived neurotrophic factor (BDNF, Peprotech) and penicillin/ streptomycin. These mixed ventral horn cultures were maintained under standard culture conditions (37°C, 5% CO_2_). All above reagents were sourced from Thermo Fisher Scientific unless otherwise specified.

### Statistical analyses

Data collection and analysis were performed blind to the conditions of the experiments. Two-tailed unpaired Student *t* test with Bonferroni corrected and 1-way ANOVA were used for the differences among multiple groups according to the number of samples, except g-ratio analysis in which Welch *t* test was used. Values of *P* < 0.05 were considered to represent a significant difference. Statistical tests were run in GraphPad Prism version 8.0 (GraphPad Software, San Diego, California, USA). Data are presented as mean ± SEM unless otherwise specified.

## Supporting information

S1 Fig Cell specificity of Syn1Cre. (**A**) Olig2 is present in certain brainstem neurons. *Syn1Cre* mice were crossed with the reporter line named “TRAP” expressing a GFP-tagged L10a ribosomal protein that is activated only in the presence of CRE [24]. Immunohistochemistry on cryo-sectioned brains from 2-month-old *Syn1Cre;TRAP* mice with cell type–specific markers such as Olig2 (OL lineage cells) and NeuN (neurons) reveals that approximately 15% of TRAP-GFP colocalized with Olig2 positive neurons, in line with previous reports that Olig2 is also expressed in a subset of neurons [25]. Scale bar = 20 μm. (**B**) The *Syn1Cre* line was crossed with the tdTomato mice and immunostained with cell-specific markers. The tdTomato was not or barely colocalized with CC1, MBP, CNPase, IBA1 nor GFAP, but only with NeuN. Blue; DAPI stained. Scale bar = 100 μm. OL, oligodendrocyte; MBP, myelin basic protein; TRAP, Translating Ribosome Affinity Purification.
(TIFF)

S2 Fig *HB9Cre* is expressed in MNs. (**A**) Immunofluorescence of *HB9-Cre; tdTomato* signal and MN marker ChAT. Representative sections from the hypoglossal nucleus (CN XII), cervical spinal cord and lumbar spinal cord are depicted. Scale bar = 50 μm. (**B**) Quantification of recombination efficiency in MNs from regions of (**A**), indicating that Cre recombination occurs highly in all MNs. *N* = 3. All data are presented as mean values +*/−* SEM. MN, motor neuron.
(TIFF)

S3 Fig Syn1Cre is active in axons of sciatic nerve. (**A**) Immunohistochemistry on P90 sciatic nerves from *Syn1Cre; tdTomato* reporter mice shows that Cre is expressed mostly in axons (Tuj1), but not in myelin (MBP). Scale bars = 100 μm and 20 μm, respectively. (**B**) Quantification of colocalized tdTomato in MBP+ or Tuj1+ cells reveals that the majority of tdTomato at P90 sciatic nerve is expressed in neurons. *N* = 3. All data are presented as mean values +*/−* SEM. Two-way ANOVA with Tukey multiple comparison tests were used. *P < 0.05, **P < 0.01 and ***P < 0.001. ns, not significant. (**C**) Representative semi-thin images of unmyelinated axons in the sciatic nerves of *Syn1Cre; Galc flox/−*. The underlying data for **B** can be found in S1 Data. GALC, galactosylceramidase; MBP, myelin basic protein.
(TIFF)

S4 Fig Gliosis is not shown in the brains of the induced neuronal *Galc* KO. Tamoxifen was injected into *Thy1-Cre/ERT2; Galc flox/−* at P2-P5, and the mice were analyzed at 2 months and 6 months old. Analysis of CD68 (red), a microgliosis marker, on the cryosections reveals that induced neuronal *Galc* KO did not activate gliosis. Scale bar = 100 μm. GALC, galactosylceramidase; KO, knockout.
(TIFF)

S5 Fig GalCer is present in neurons. O1-specific antibody that detects GalCer (green) was colocalized (arrows) with neuronal marker protein, Npas4 (red), in the brain. GalCer, galactosylceramide.
(TIFF)

S6 Fig Comparison of myelin proteins and GFAP between haplodeficient *Galc +/−* and *Galc flox/−* or *Galc +/+*. (**A**) Western blot analysis of total brain extracts shows that the haplodeficient *Galc* +*/−* did not affect the levels of myelin proteins and GFAP, compared to *Galc +/+* WT. (**B**) The expression of myelin proteins such as MAG and MBP was not different between both haplodeficient *Galc +/−* and *Galc flox/−*. *N* = 3. The underlying data for **B** can be found in S1 Data. GALC, galactosylceramidase; MAG, myelin-associated glycoprotein; MBP, myelin basic protein; WT, wild-type.
(TIFF)

S1 Data Numerical values used for plots and statistical analysis. (XLSX)

S1 Raw images Original western blot membrane images. (PDF)

## Abbreviations

AMPA: α-amino-3-hydroxy-5-methyl-4-isoxazolepropionic acid
BDNF: brain-derived neurotrophic factor
CNS: central nervous system
CNTF: ciliary neurotrophic factor
GALC: galactosylceramidase
GalCer: galactosylceramide
GBA: glucocerebrosidase
KO: knockout
LC–MS–MS: high-performance liquid chromatography tandem mass spectrometry
LiBH4: lithium borohydride
MAG: myelin-associated glycoprotein
MBP: myelin basic protein
MN: motor neuron
MRM: multiple reaction monitoring
OL: oligodendrocyte
PLP: proteolipid protein
PNS: peripheral nervous system
RIPA: Radio-Immuno-Precipitation Assay
SPH: Synaptophysin
TRAP: Translating Ribosome Affinity Purification
Tbr1: T-brain-1
WT: wild-type

## References

[1] Suzuki K, Suzuki Y. Globoid cell leucodystrophy (Krabbe’s disease): deficiency of galactocerebroside beta-galactosidase. Proc Natl Acad Sci U S A. 1970;66(2):302–9. Epub 1970/06/01. doi: 10.1073/pnas.66.2.302 ; PubMed Central PMCID: PMC283044. 5271165 PMC283044

[2] Li Y, Xu Y, Benitez BA, Nagree MS, Dearborn JT, Jiang X, et al. Genetic ablation of acid ceramidase in Krabbe disease confirms the psychosine hypothesis and identifies a new therapeutic target. Proc Natl Acad Sci U S A. 2019;116(40):20097–103. doi: 10.1073/pnas.1912108116 31527255 PMC6778236

[3] Miyatake T, Suzuki K. Globoid cell leukodystrophy: Additional deficiency of psychosine galactosidase. Biochem Biophys Res Commun. 1972;48(3):538–43. doi: 10.1016/0006-291x(72)90381-6 5047687

[4] Suzuki K. Twenty Five Years of the “Psychosine Hypothesis”: A Personal Perspective of its History and Present Status. Neurochem Res. 1998;23(3):251–9. doi: 10.1023/a:1022436928925 9482237

[5] White AB, Givogri MI, Lopez-Rosas A, Cao H, Breemen Rv, Thinakaran G, et al. Psychosine Accumulates in Membrane Microdomains in the Brain of Krabbe Patients, Disrupting the Raft Architecture. J Neurosci. 2009;29(19): 6068–77. doi: 10.1523/JNEUROSCI.5597-08.2009 19439584 PMC6665501

[6] Formichi P, Radi E, Battisti C, Pasqui A, Pompella G, Lazzerini PE, et al. Psychosine-induced apoptosis and cytokine activation in immune peripheral cells of Krabbe patients. J Cell Physiol. 2007;212(3):737–43. Epub 2007/04/27. doi: 10.1002/jcp.21070 . 17458901

[7] Tanaka K, Webster HD. Effects of psychosine (galactosylsphingosine) on the survival and the fine structure of cultured Schwann cells. J Neuropathol Exp Neurol. 1993;52(5):490–8. Epub 1993/09/01. doi: 10.1097/00005072-199309000-00007 . 8360702

[8] Folts CJ, Scott-Hewitt N, Proschel C, Mayer-Proschel M, Noble M. Lysosomal Re-acidification Prevents Lysosphingolipid-Induced Lysosomal Impairment and Cellular Toxicity. PLoS Biol. 2016;14(12):e1002583. Epub 2016/12/16. doi: 10.1371/journal.pbio.1002583 ; PubMed Central PMCID: PMC5169359. 27977664 PMC5169359

[9] Zaka M, Wenger DA. Psychosine-induced apoptosis in a mouse oligodendrocyte progenitor cell line is mediated by caspase activation. Neurosci Lett. 2004;358(3):205–9. Epub 2004/03/25. doi: 10.1016/j.neulet.2003.12.126 . 15039117

[10] Mikoshiba K, Fujishiro M, Kohsaka S, Okano H, Takamatsu K, Tsukada Y. Disorders in myelination in the twitcher mutant: immunohistochemical and biochemical studies. Neurochem Res. 1985;10(8):1129–41. Epub 1985/08/01. doi: 10.1007/BF00965887 . 2414681

[11] Suzuki K. Globoid cell leukodystrophy (Krabbe’s disease): update. J Child Neurol. 2003;18(9):595–603. Epub 2003/10/24. doi: 10.1177/08830738030180090201 . 14572137

[12] Taniike M, Suzuki K. Spacio-temporal progression of demyelination in twitcher mouse: with clinico-pathological correlation. Acta Neuropathol. 1994;88(3):228–36. Epub 1994/01/01. doi: 10.1007/BF00293398 . 7528964

[13] Tanaka K, Nagara H, Kobayashi T, Goto I. The twitcher mouse: accumulation of galactosylsphingosine and pathology of the sciatic nerve. Brain Res. 1988;454(1–2):340–6. Epub 1988/06/28. doi: 10.1016/0006-8993(88)90835-9 . 3409017

[14] Suzuki K. Krabbe Disease: Myelin Biology and Disorders. USA: Elsevier; 2004. 841–50 p.

[15] Feltri ML, Weinstock NI, Favret J, Dhimal N, Wrabetz L, Shin D. Mechanisms of demyelination and neurodegeneration in globoid cell leukodystrophy. Glia. 2021. doi: 10.1002/glia.24008 33851745 PMC8502241

[16] Castelvetri LC, Givogri MI, Zhu H, Smith B, Lopez-Rosas A, Qiu X, et al. Axonopathy is a compounding factor in the pathogenesis of Krabbe disease. Acta Neuropathol. 2011;122(1):35–48. doi: 10.1007/s00401-011-0814-2 21373782 PMC3690521

[17] Weinstock NI, Kreher C, Favret J, Nguyen D, Bongarzone ER, Wrabetz L, et al. Brainstem development requires galactosylceramidase and is critical for pathogenesis in a model of Krabbe disease. Nat Comm. 2020;11(1):5356. 10.1038/s41467-020-19179-w PMC7584660 33097716

[18] Dolcetta D, Perani L, Givogri MI, Galbiati F, Orlacchio A, Martino S, et al. Analysis of Galactocerebrosidase Activity in the Mouse Brain by a New Histological Staining Method. J Neurosci Res. 2004;77:462–4. doi: 10.1002/jnr.20169 15248301

[19] Zhang Y, Chen K, Sloan SA, Bennett ML, Scholze AR, O’Keeffe S, et al. An RNA-Sequencing Transcriptome and Splicing Database of Glia, Neurons, and Vascular Cells of the Cerebral Cortex. J Neurosci. 2014;34(36):11929–47. doi: 10.1523/JNEUROSCI.1860-14.2014 25186741 PMC4152602

[20] Teixeira CA, Miranda CO, Sousa VF, Santosa TE, Malheiro AR, Solomon M, et al. Early axonal loss accompanied by impaired endocytosis, abnormal axonal transport, and decreased microtubule stability occur in the model of Krabbe’s disease. Neurobiology of Disease. 2014;66:92–103. doi: 10.1016/j.nbd.2014.02.012 24607884 PMC4307018

[21] Lim SM, Choi B-O, Oh S-i, Choi WJ, Oh K-W, Nahm M, et al. Patient fibroblasts-derived induced neurons demonstrate autonomous neuronal defects in adult-onset Krabbe disease. Oncotarget. 2016;7(46):74496–509. doi: 10.18632/oncotarget.12812 27780934 PMC5342682

[22] Hoesche C, Sauerwald A, Veh RW, Krippl B, Kilimann MW. The 5’-flanking region of the rat synapsin I gene directs neuron-specific and developmentally regulated reporter gene expression in transgenic mice. J Biol Chem. 1993;268(35):26494–502. Epub 1993/12/15. . 8253778

[23] Zhu Y, Romero MI, Ghosh P, Ye Z, Charnay P, Rushing EJ, et al. Ablation of NF1 function in neurons induces abnormal development of cerebral cortex and reactive gliosis in the brain. Genes Dev. 2001;15(7):859–76. doi: 10.1101/gad.862101 11297510 PMC312666

[24] Liu J, Krautzberger AM, Sui SH, Hofmann OM, Chen Y, Baetscher M, et al. Cell-specific translational profiling in acute kidney injury. J Clin Invest. 2014;124(3):1242–54. Epub 2014/02/27. doi: 10.1172/JCI72126 ; PubMed Central PMCID: PMC3938273. 24569379 PMC3938273

[25] Sagner A, Gaber ZB, Delile J, Kong JH, Rousso DL, Pearson CA, et al. Olig2 and Hes regulatory dynamics during motor neuron differentiation revealed by single cell transcriptomics. PLoS Biol. 2018;16(2):e2003127. doi: 10.1371/journal.pbio.2003127 29389974 PMC5811045

[26] Madisen L, Zwingman TA, Sunkin SM, Oh SW, Zariwala HA, Gu H, et al. A robust and high-throughput Cre reporting and characterization system for the whole mouse brain. Nat Neurosci. 2010;13(1):133–40. doi: 10.1038/nn.2467 20023653 PMC2840225

[27] Foran DR, Peterson AC. Myelin acquisition in the central nervous system of the mouse revealed by an MBP-Lac Z transgene. J Neurosci. 1992;12(12):4890–7. Epub 1992/12/01. doi: 10.1523/JNEUROSCI.12-12-04890.1992 ; PubMed Central PMCID: PMC6575777. 1281497 PMC6575777

[28] Mendes CS, Bartos I, Marka Z, Akay T, Marka S, Mann RS. Quantification of gait parameters in freely walking rodents. BMC Biol. 2015;13:50. Epub 2015/07/23. doi: 10.1186/s12915-015-0154-0 ; PubMed Central PMCID: PMC4511453. 26197889 PMC4511453

[29] Muthusamy K, Sudhakar SV, Thomas M, Yoganathan S, Christudass CS, Chandran M, et al. Revisiting magnetic resonance imaging pattern of Krabbe disease—Lessons from an Indian cohort. J Clin Imaging Sci. 2019;9:25. Epub 2019/08/27. doi: 10.25259/JCIS-18-2019 ; PubMed Central PMCID: PMC6702867. 31448176 PMC6702867

[30] Weiss T, Miltner WH, Liepert J, Meissner W, Taub E. Rapid functional plasticity in the primary somatomotor cortex and perceptual changes after nerve block. Eur J Neurosci. 2004;20(12):3413–23. Epub 2004/12/22. doi: 10.1111/j.1460-9568.2004.03790.x . 15610174

[31] Byun N, Delpire E. Axonal and periaxonal swelling precede peripheral neurodegeneration in KCC3 knockout mice. Neurobiol Dis. 2007;28(1):39–51. doi: 10.1016/j.nbd.2007.06.014 17659877 PMC2242858

[32] Braak E, Sandmann-Keil D, Rüb U, Gai WP, de Vos RA, Steur EN, et al. alpha-synuclein immunopositive Parkinson’s disease-related inclusion bodies in lower brain stem nuclei. Acta Neuropathol. 2001;101(3):195–201. Epub 2001/04/20. doi: 10.1007/s004010000247 . 11307617

[33] Potter GB, Santos M, Davisson MT, Rowitch DH, Marks DL, Bongarzone ER, et al. Missense mutation in mouse GALC mimics human gene defect and offers new insights into Krabbe disease. Hum Mol Genet. 2013;22(17):3397–414. doi: 10.1093/hmg/ddt190 23620143 PMC3736866

[34] Porrero C, Rubio-Garrido P, Avendaño C, Clascá F. Mapping of fluorescent protein-expressing neurons and axon pathways in adult and developing Thy1-eYFP-H transgenic mice. Brain Research. 2010;1345:59–72. doi: 10.1016/j.brainres.2010.05.061 20510892

[35] Hevner RF, Shi L, Justice N, Hsueh Y-P, Sheng M, Smiga S, et al. Tbr1 Regulates Differentiation of the Preplate and Layer 6. Neuron. 2001;29(2):353–66. doi: 10.1016/s0896-6273(01)00211-2 11239428

[36] Xia Yang, Arber S, William C, Li L, Tanabe Y, Jessell TM, et al. Patterning of Muscle Acetylcholine Receptor Gene Expression in the Absence of Motor Innervation. Neuron. 2001;30(2):399–410. doi: 10.1016/s0896-6273(01)00287-2 11395002

[37] D’Auria L, Reiter C, Ward E, Moyano AL, Marshall MS, Nguyen D, et al. Psychosine enhances the shedding of membrane microvesicles: Implications in demyelination in Krabbe’s disease. PLoS ONE. 2017;12(5):e0178103. Epub 2017/05/23. doi: 10.1371/journal.pone.0178103 ; PubMed Central PMCID: PMC5439731. 28531236 PMC5439731

[38] White AB, Galbiati F, Givogri MI, Lopez Rosas A, Qiu X, van Breemen R, et al. Persistence of psychosine in brain lipid rafts is a limiting factor in the therapeutic recovery of a mouse model for Krabbe disease. J Neurosci Res. 2011;89(3):352–64. Epub 2011/01/25. doi: 10.1002/jnr.22564 ; PubMed Central PMCID: PMC3064524. 21259322 PMC3064524

[39] Zulueta Diaz YLM, Caby S, Bongarzone ER, Fanani ML. Psychosine remodels model lipid membranes at neutral pH. Biochim Biophys Acta Biomembr. 2018;1860(12):2515–26. Epub 2018/09/30. doi: 10.1016/j.bbamem.2018.09.015 . 30267657

[40] Weinstock N, Shin D, Dhimal N, Hong X, Irons EE, Silvestri NJ, et al. Macrophages Expressing GALC Improve Peripheral Krabbe Disease by a Mechanism Independent of Cross-Correction. Neuron. 2020;107(1):65–81. doi: 10.1016/j.neuron.2020.03.031 32375064 PMC7924901

[41] Cappello V, Marchetti L, Parlanti P, Landi S, Tonazzini I, Cecchini M, et al. Ultrastructural Characterization of the Lower Motor System in a Mouse Model of Krabbe Disease. Sci Rep. 2016;6(1):1. doi: 10.1038/s41598-016-0001-8 28442746 PMC5431369

[42] Reiter CR, Rebiai R, Kwak A, Marshall J, Wozniak D, Scesa G, et al. The Pathogenic Sphingolipid Psychosine is Secreted in Extracellular Vesicles in the Brain of a Mouse Model of Krabbe Disease. ASN Neuro. 2022;14:17590914221087817. Epub 2022/03/19. doi: 10.1177/17590914221087817 . 35300522 PMC8943320

[43] Mitew S, Gobius I, Fenlon LR, McDougall SJ, Hawkes D, Xing YL, et al. Pharmacogenetic stimulation of neuronal activity increases myelination in an axon-specific manner. Nat Comm. 2018;9(1):306. 10.1038/s41467-017-02719-2 PMC5778130 29358753

[44] Barres BA, Schmid R, Sendnter M, Raff MC. Multiple extracellular signals are required for long-term oligodendrocyte survival. Development. 1993;118(1):283–95. Epub 1993/05/01. doi: 10.1242/dev.118.1.283 . 8375338

[45] Taveggia C, Feltri ML, Wrabetz L. Signals to promote myelin formation and repair. Nat Rev Neurol. 2010;6(5):276–87. Epub 2010/04/21. doi: 10.1038/nrneurol.2010.37 ; PubMed Central PMCID: PMC3062363. 20404842 PMC3062363

[46] Duncan GJ, Simkins TJ, Emery B. Neuron-Oligodendrocyte Interactions in the Structure and Integrity of Axons. Frontiers in Cell and Developmental Biology. 2021;9(460). doi: 10.3389/fcell.2021.653101 33763430 PMC7982542

[47] Foster LJ, De Hoog CL, Mann M. Unbiased quantitative proteomics of lipid rafts reveals high specificity for signaling factors. Proc Natl Acad Sci U S A. 2003;100(10):5813–8. Epub 2003/05/02. doi: 10.1073/pnas.0631608100 ; PubMed Central PMCID: PMC156283. 12724530 PMC156283

[48] Tsui-Pierchala BA, Encinas M, Milbrandt J, Johnson EM Jr., Lipid rafts in neuronal signaling and function. Trends Neurosci. 2002;25(8):412–7. Epub 2002/07/20. doi: 10.1016/s0166-2236(02)02215-4 . 12127758

[49] Asotra K, Macklin WB. Protein kinase C activity modulates myelin gene expression in enriched oligodendrocytes. J Neurosci Res. 1993;34(5):571–88. Epub 1993/04/01. doi: 10.1002/jnr.490340509 . 7683060

[50] Tanaka C, Nishizuka Y. The protein kinase C family for neuronal signaling. Annu Rev Neurosci. 1994;17:551–67. Epub 1994/01/01. doi: 10.1146/annurev.ne.17.030194.003003 . 8210187

[51] Cantuti-Castelvetri L, Zhu H, Givogri MI, Chidavaenzi RL, Lopez-Rosas A, Bongarzone ER. Psychosine induces the dephosphorylation of neurofilaments by deregulation of PP1 and PP2A phosphatases. Neurobiol Dis. 2012;46(2):325–35. Epub 2012/02/14. doi: 10.1016/j.nbd.2012.01.013 ; PubMed Central PMCID: PMC3323754. 22326830 PMC3323754

[52] Cantuti Castelvetri L, Givogri MI, Hebert A, Smith B, Song Y, Kaminska A, et al. The sphingolipid psychosine inhibits fast axonal transport in Krabbe disease by activation of GSK3beta and deregulation of molecular motors. J Neurosci. 2013;33(24):10048–56. Epub 2013/06/14. doi: 10.1523/JNEUROSCI.0217-13.2013 ; PubMed Central PMCID: PMC3682375. 23761900 PMC3682375

[53] Suzuki K, Tanaka H, Suzuki K. Studies on the pathogenesis of Krabbe’s leukodystrophy: cellular reaction of the brain to exogenous galactosylsphingosine, monogalactosyl diglyceride, and lactosylceramide. Adv Exp Med Biol. 1976;68:99–114. Epub 1976/01/01. doi: 10.1007/978-1-4684-7735-1_7 . 937124

[54] Zhou T, Zheng Y, Sun L, Badea SR, Jin Y, Liu Y, et al. Microvascular endothelial cells engulf myelin debris and promote macrophage recruitment and fibrosis after neural injury. Nat Neurosci. 2019;22(3):421–35. Epub 2019/01/22. doi: 10.1038/s41593-018-0324-9 ; PubMed Central PMCID: PMC6913093. 30664769 PMC6913093

[55] Pfisterer U, Khodosevich K. Neuronal survival in the brain: neuron type-specific mechanisms. Cell Death Dis. 2017;8(3):e2643. doi: 10.1038/cddis.2017.64 28252642 PMC5386560

[56] Schaeren-Wiemers N, Bijl Pvd, Schwab ME. The UDP-galactose:ceramide galactosyltransferase: expression pattern in oligodendrocytes and Schwann cells during myelination and substrate preference for hydroxyceramide. J Neurochem. 1995;65(5):2267–78. doi: 10.1046/j.1471-4159.1995.65052267.x 7595516

[57] Persaud-Sawin D-A, II JOM, Rylova S, Vandongen A, Boustany R-MN. A Galactosylceramide Binding Domain Is Involved in Trafficking of CLN3 from Golgi to Rafts via Recycling Endosomes. Pediatr Res. 2004;56:449–63. doi: 10.1203/01.PDR.0000136152.54638.95 15240864

[58] Chamberlain KA, Huang N, Xie Y, LiCausi F, Li S, Li Y, et al. Oligodendrocytes enhance axonal energy metabolism by deacetylation of mitochondrial proteins through transcellular delivery of SIRT2. Neuron. 2021;109(21):3456–72.e8. doi: 10.1016/j.neuron.2021.08.011 34506725 PMC8571020

[59] Moreno-García A, Kun A, Calero O, Medina M, Calero M. An Overview of the Role of Lipofuscin in Age-Related Neurodegeneration. Frontiers in Neuroscience. 2018;12. doi: 10.3389/fnins.2018.00464 30026686 PMC6041410

[60] Chang D, Nalls MA, Hallgrímsdóttir IB, Hunkapiller J, van der Brug M, Cai F, et al. A meta-analysis of genome-wide association studies identifies 17 new Parkinson’s disease risk loci. Nat Genet. 2017;49(10):1511–6. Epub 2017/09/12. doi: 10.1038/ng.3955 ; PubMed Central PMCID: PMC5812477. 28892059 PMC5812477

[61] Riboldi GM, Fonzo ABD. GBA, Gaucher Disease, and Parkinson’s Disease: From Genetic to Clinic to New Therapeutic Approaches. Cells. 2019;8(4):364. 10.3390/cells8040364 PMC6523296 31010158

[62] Marshall MS, Bongarzone ER. Beyond Krabbe’s disease: the potential contribution of GALC deficiency to neuronal vulnerability in late onset synucleinopathies. J Neurosci Res. 2016;94(11):1328–32. doi: 10.1002/jnr.23751 27638614 PMC5027968

[63] Cantuti-Castelvetri L, Bongarzone ER. SYNAPTIC FAILURE: THE ACHILLES TENDON OF SPHINGOLIPIDOSES. J Neurosci Res. 2016;(11):1031–6. doi: 10.1002/jnr.23753 27638588 PMC5027974

[64] Smith BR, Santos MB, Marshall MS, Cantuti-Castelvetri L, Lopez-Rosas A, Li G, et al. Neuronal inclusions of α-synuclein contribute to the pathogenesis of Krabbe disease. J Pathol. 2014;232(5):509–21. doi: 10.1002/path.4328 24415155 PMC3977150

[65] Hensch TK. Critical period plasticity in local cortical circuits. Nat Rev Neurosci. 2005;6:877–88. doi: 10.1038/nrn1787 16261181

[66] Marín O. Developmental timing and critical windows for the treatment of psychiatric disorders. Nat Medicine. 2016;22:1229–38. doi: 10.1038/nm.4225 27783067

[67] Vukoja A, Rey U, Petzoldt AG, Ott C, Vollweiter D, Quentin C, et al. Presynaptic Biogenesis Requires Axonal Transportof Lysosome-Related Vesicles. Neuron. 2018;99:1216–32. doi: 10.1016/j.neuron.2018.08.004 30174114

[68] Goo MS, Sancho L, Slepak N, Boassa D, Deerinck TJ, Ellisman MH, et al. Activity-dependent trafficking of lysosomes in dendrites and dendritic spines. J Cell Biol. 2017;216(8). doi: 10.1083/jcb.201704068 28630145 PMC5551717

[69] Padamsey Z, McGuinness L, Bardo SJ, Reinhart M, Tong R, Hedegaard A, et al. Activity-Dependent Exocytosis of Lysosomes Regulates the Structural Plasticity of Dendritic Spines. Neuron. 2017;93(1):132–46. Epub 2016/12/19. doi: 10.1016/j.neuron.2016.11.013 ; PubMed Central PMCID: PMC5222721. 27989455 PMC5222721

[70] Fernández-Monreal M, Brown TC, Royo M, Esteban JA. The balance between receptor recycling and trafficking toward lysosomes determines synaptic strength during long-term depression. J Neurosci. 2012;32(38):13200–5. doi: 10.1523/JNEUROSCI.0061-12.2012 22993436 PMC6621469

[71] Calderon RO, Attema B, DeVries GH. Lipid composition of neuronal cell bodies and neurites from cultured dorsal root ganglia. J Neurochem. 1995;64(1):424–9. Epub 1995/01/01. doi: 10.1046/j.1471-4159.1995.64010424.x . 7798942

[72] van Echten-Deckert G, Herget T. Sphingolipid metabolism in neural cells. Biochim Biophys Acta. 2006;1758(12):1978–94. Epub 2006/07/18. doi: 10.1016/j.bbamem.2006.06.009 . 16843432

[73] LeVine S, Wetzel D, Eilert A. Neuropathology of twitcher mice: examination by histochemistry, immunohistochemistry, lectin histochemistry and Fourier transform infrared microspectroscopy. Int J Dev Neurosci. 1994;12(4):275–88. doi: 10.1016/0736-5748(94)90075-2 7526605

[74] Schafer DP, Stevens B. Phagocytic glial cells: sculpting synaptic circuits in the developing nervous system. Curr Opin Neurobiol. 2013;23(6):1034–40. Epub 2013/10/26. doi: 10.1016/j.conb.2013.09.012 ; PubMed Central PMCID: PMC3907950. 24157239 PMC3907950

[75] Kim EM, Hwang O. Role of matrix metalloproteinase-3 in neurodegeneration. J Neurochem. 2011;116(1):22–32. Epub 2010/11/04. doi: 10.1111/j.1471-4159.2010.07082.x . 21044079

[76] Sokolova A, Hill MD, Rahimi F, Warden LA, Halliday GM, Shepherd CE. Monocyte chemoattractant protein-1 plays a dominant role in the chronic inflammation observed in Alzheimer’s disease. Brain Pathol. 2009;19(3):392–8. Epub 2008/07/19. doi: 10.1111/j.1750-3639.2008.00188.x . 18637012 PMC8094842

[77] Rempe D, Vangeison G, Hamilton J, Li Y, Jepson M, Federoff H. Synapsin I Cre transgene expression in male mice produces germline recombination in progeny. Genesis. 2006;44(1):44–9. doi: 10.1002/gene.20183 16419044

[78] Lee WC, Kang D, Causevic E, Herdt AR, Eckman EA, Eckman CB. Molecular characterization of mutations that cause globoid cell leukodystrophy and pharmacological rescue using small molecule chemical chaperones. J Neurosci. 2010;30(16):5489–97. Epub 2010/04/23. doi: 10.1523/JNEUROSCI.6383-09.2010 ; PubMed Central PMCID: PMC3278277. 20410102 PMC3278277

[79] Kun A, González-Camacho F, Hernández S, Moreno-García A, Calero O, Calero M. Characterization of Amyloid-β Plaques and Autofluorescent Lipofuscin Aggregates in Alzheimer’s Disease Brain: A Confocal Microscopy Approach. In: Sigurdsson EM, Calero M, Gasset M, editors. Amyloid Proteins: Methods and Protocols. New York, NY: Springer New York; 2018. p. 497–512. 10.1007/978-1-4939-7816-8_31 29886553

[80] Berg S, Kutra D, Kroeger T, Straehle CN, Kausler BX, Haubold C, et al. ilastik: interactive machine learning for (bio)image analysis. Nat Methods. 2019;16(12):1226–32. Epub 2019/10/02. doi: 10.1038/s41592-019-0582-9 . 31570887

[81] Heiman M, Kulicke R, Fenster RJ, Greengard P, Heintz N. Cell type–specific mRNA purification by translating ribosome affinity purification (TRAP). Nat Protoc. 2014;9(6):1282–91. doi: 10.1038/nprot.2014.085 24810037 PMC4102313

[82] Livak KJ, Schmittgen TD. Analysis of relative gene expression data using real-time quantitative PCR and the 2(-Delta Delta C(T)) Method. Methods. 2001;25(4):402–8. Epub 2002/02/16. doi: 10.1006/meth.2001.1262 . 11846609

[83] Shin D, Feltri ML, Wrabetz L. Altered Trafficking and Processing of GALC Mutants Correlates with Globoid Cell Leukodystrophy Severity. J Neurosci. 2016;36(6):1858–70. Epub 2016/02/13. doi: 10.1523/JNEUROSCI.3095-15.2016 ; PubMed Central PMCID: PMC4748072. 26865610 PMC4748072

[84] Liao H-C, Chan M-J, Yang C-F, Chiang C-C, Niu D-M, Huang C-K, et al. Mass Spectrometry but Not Fluorometry Distinguishes Affected and Pseudodeficiency Patients in Newborn Screening for Pompe Disease. Clin Chem. 2017;In Press. 10.1373/clinchem.2016.269027 PMC5524447 28450385

[85] Henderson CE, Bloch-Gallego E, Camu W. Purified embryonic motoneurons. In: Cohen J, Wilkin G, editors. Nerve Cell Cultures: A Practical Approach. Oxford University, London 1995. p. 69–81.

