# Supplementary figures and images for: Neuron-specific ablation of the Krabbe disease gene galactosylceramidase in mice results in neurodegeneration
Source: PLoS Biol. 2022 Jul 5;20(7):e3001661. doi: 10.1371/journal.pbio.3001661 (PMC9255775; doi:10.1371/journal.pbio.3001661)

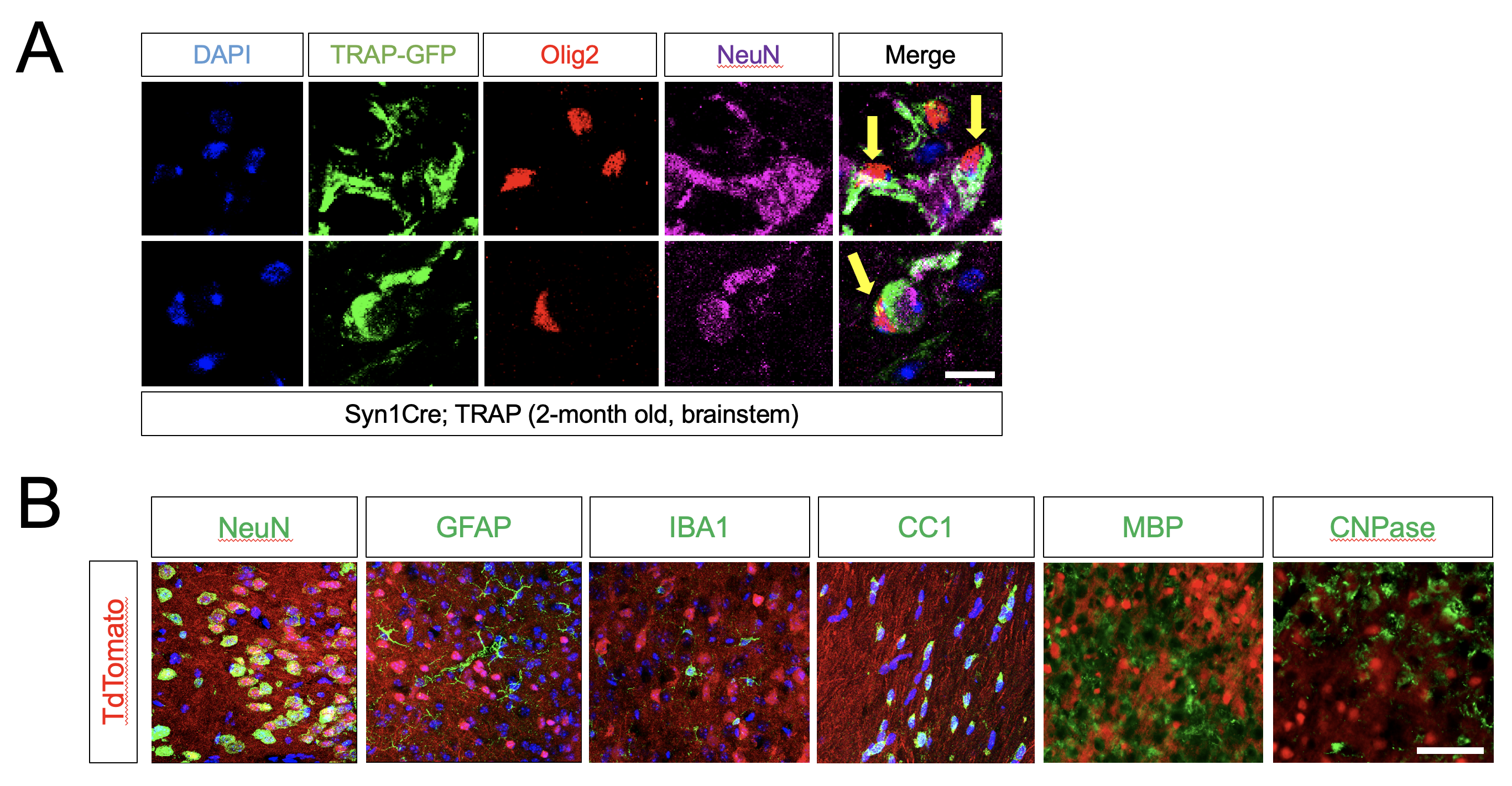

Supplement: S1 Fig — (A) Olig2 is present in certain brainstem neurons. Syn1Cre mice were crossed with the reporter line named “TRAP” expressing a GFP-tagged L10a ribosomal protein that is activated only in the presence of CRE [24]. Immunohistochemistry on cryo-sectioned brains from 2-month-old Syn1Cre;TRAP mice with cell type–specific markers such as Olig2 (OL lineage cells) and NeuN (neurons) reveals that approximately 15% of TRAP-GFP colocalized with Olig2 positive neurons, in line with previous reports that Olig2 is also expressed in a subset of neurons [25]. Scale bar = 20 μm. (B) The Syn1Cre line was crossed with the tdTomato mice and immunostained with cell-specific markers. The tdTomato was not or barely colocalized with CC1, MBP, CNPase, IBA1 nor GFAP, but only with NeuN. Blue; DAPI stained. Scale bar = 100 μm. OL, oligodendrocyte; MBP, myelin basic protein; TRAP, Translating Ribosome Affinity Purification. (TIFF) [file pbio.3001661.s001.tiff]

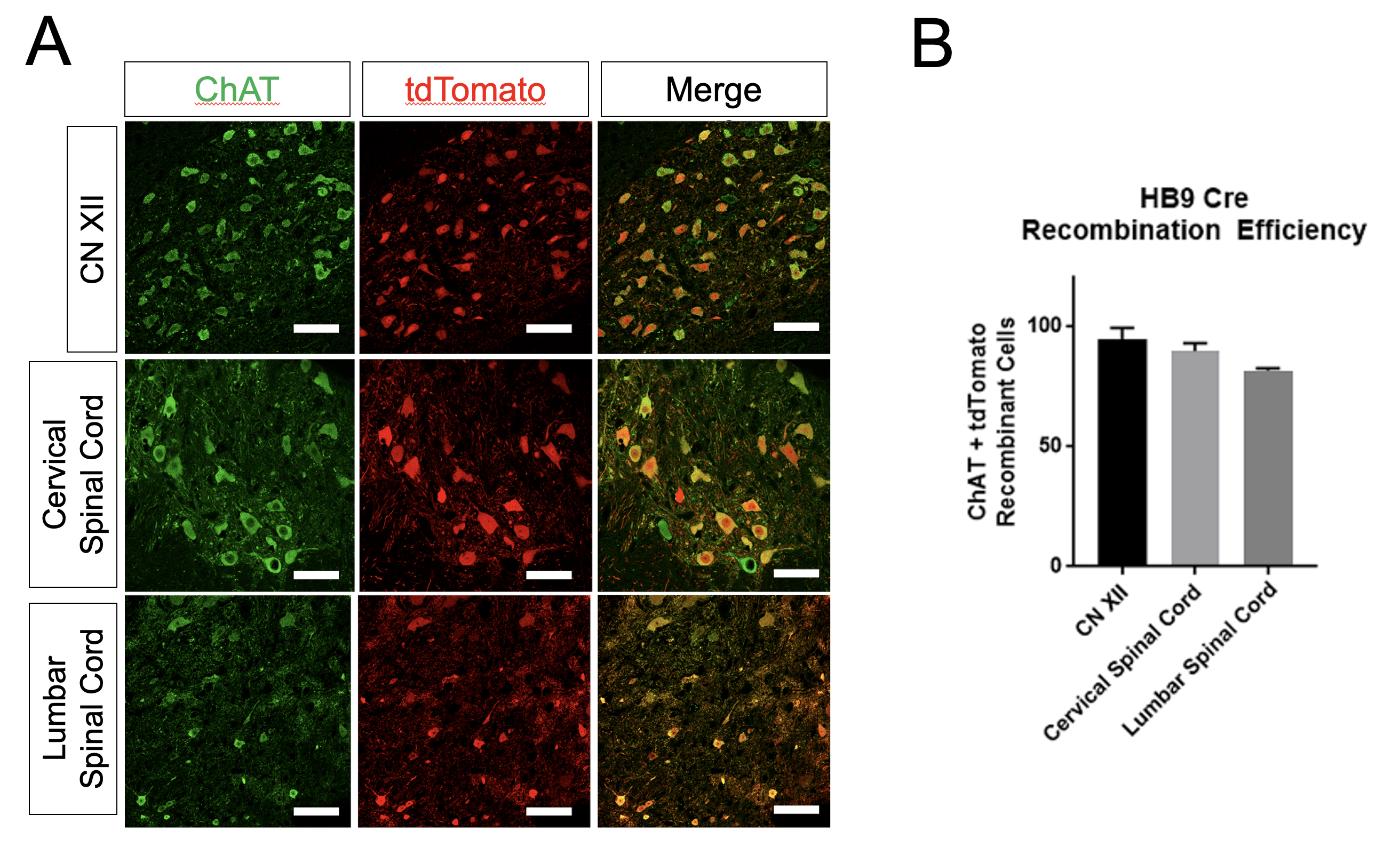

Supplement: S2 Fig — (A) Immunofluorescence of HB9-Cre; tdTomato signal and MN marker ChAT. Representative sections from the hypoglossal nucleus (CN XII), cervical spinal cord and lumbar spinal cord are depicted. Scale bar = 50 μm. (B) Quantification of recombination efficiency in MNs from regions of (A), indicating that Cre recombination occurs highly in all MNs. N = 3. All data are presented as mean values +/− SEM. MN, motor neuron. (TIFF) [file pbio.3001661.s002.tiff]

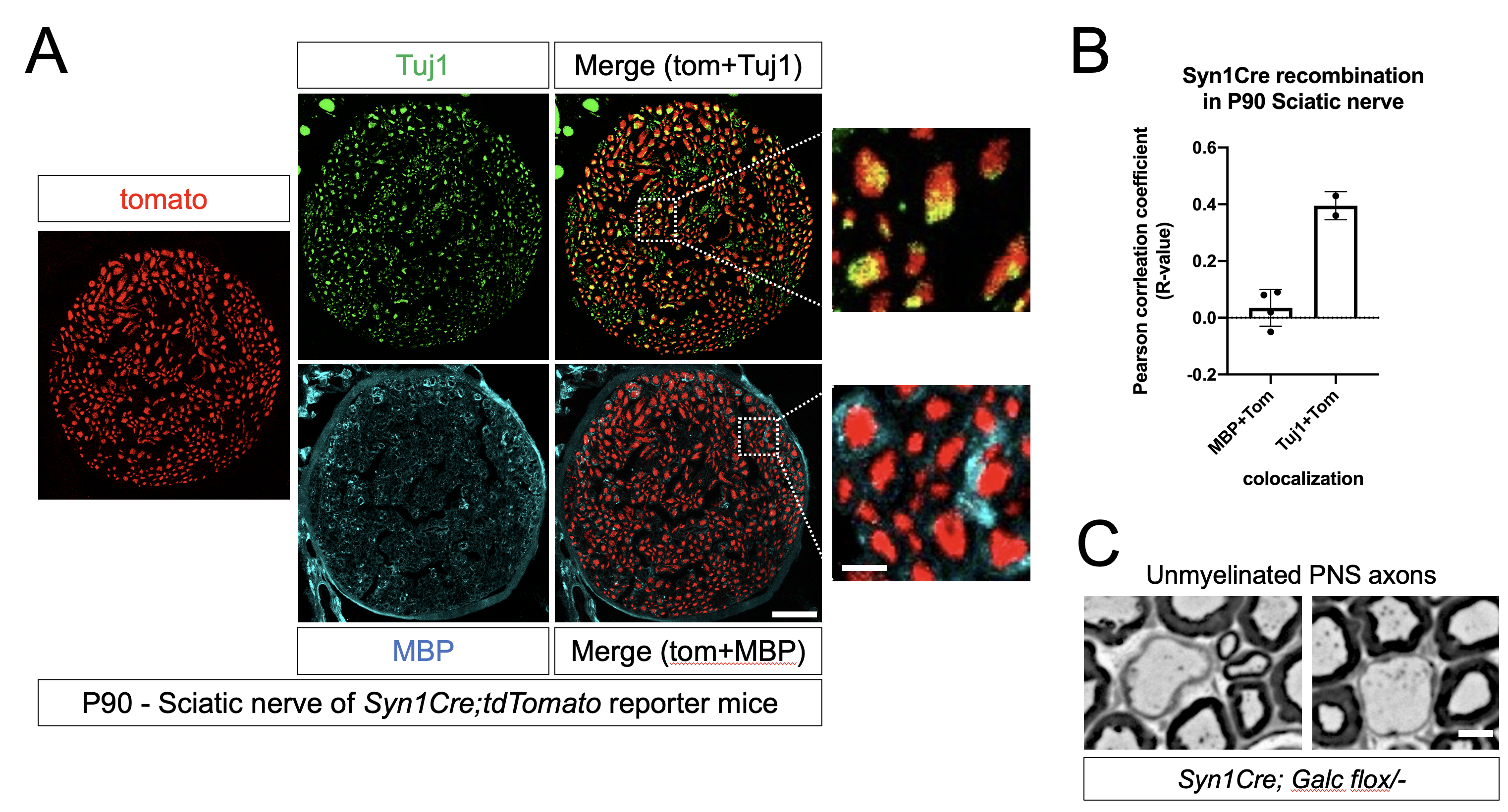

Supplement: S3 Fig — (A) Immunohistochemistry on P90 sciatic nerves from Syn1Cre; tdTomato reporter mice shows that Cre is expressed mostly in axons (Tuj1), but not in myelin (MBP). Scale bars = 100 μm and 20 μm, respectively. (B) Quantification of colocalized tdTomato in MBP+ or Tuj1+ cells reveals that the majority of tdTomato at P90 sciatic nerve is expressed in neurons. N = 3. All data are presented as mean values +/− SEM. Two-way ANOVA with Tukey multiple comparison tests were used. *P < 0.05, **P < 0.01 and ***P < 0.001. ns, not significant. (C) Representative semi-thin images of unmyelinated axons in the sciatic nerves of Syn1Cre; Galc flox/−. The underlying data for B can be found in S1 Data. GALC, galactosylceramidase; MBP, myelin basic protein. (TIFF) [file pbio.3001661.s003.tiff]

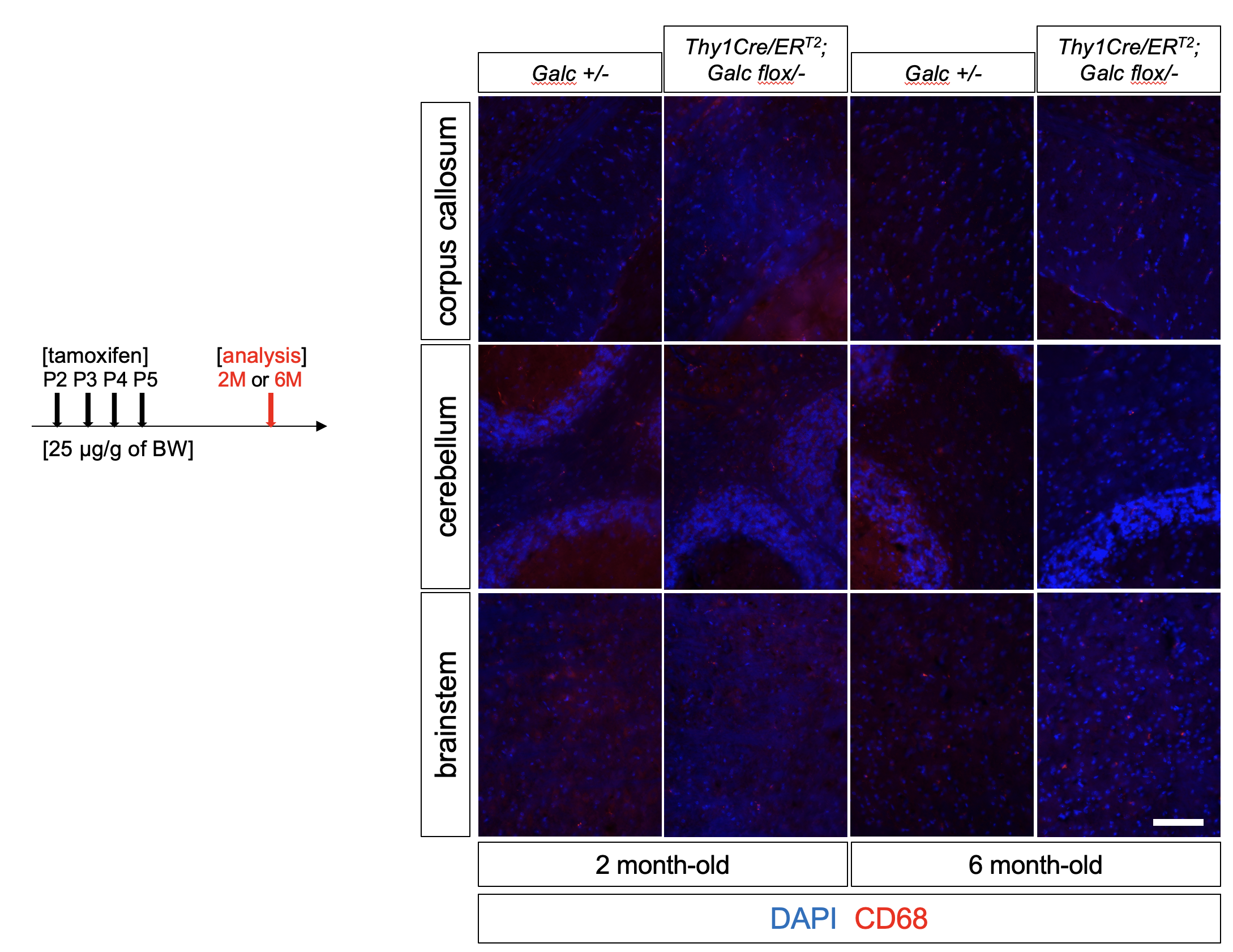

Supplement: S4 Fig — Tamoxifen was injected into Thy1-Cre/ERT2; Galc flox/− at P2-P5, and the mice were analyzed at 2 months and 6 months old. Analysis of CD68 (red), a microgliosis marker, on the cryosections reveals that induced neuronal Galc KO did not activate gliosis. Scale bar = 100 μm. GALC, galactosylceramidase; KO, knockout. (TIFF) [file pbio.3001661.s004.tiff]

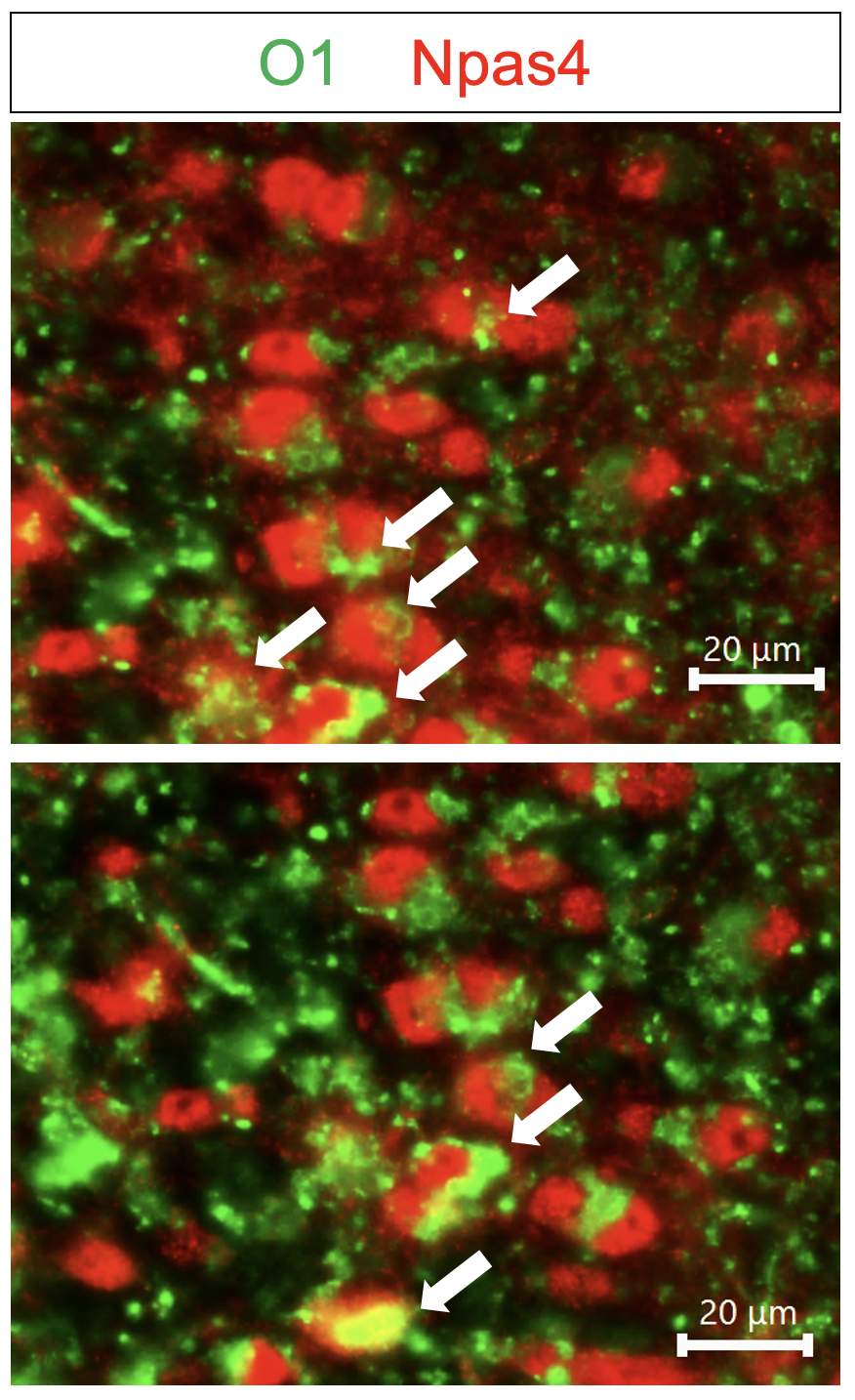

Supplement: S5 Fig — O1-specific antibody that detects GalCer (green) was colocalized (arrows) with neuronal marker protein, Npas4 (red), in the brain. GalCer, galactosylceramide. (TIFF) [file pbio.3001661.s005.tiff]

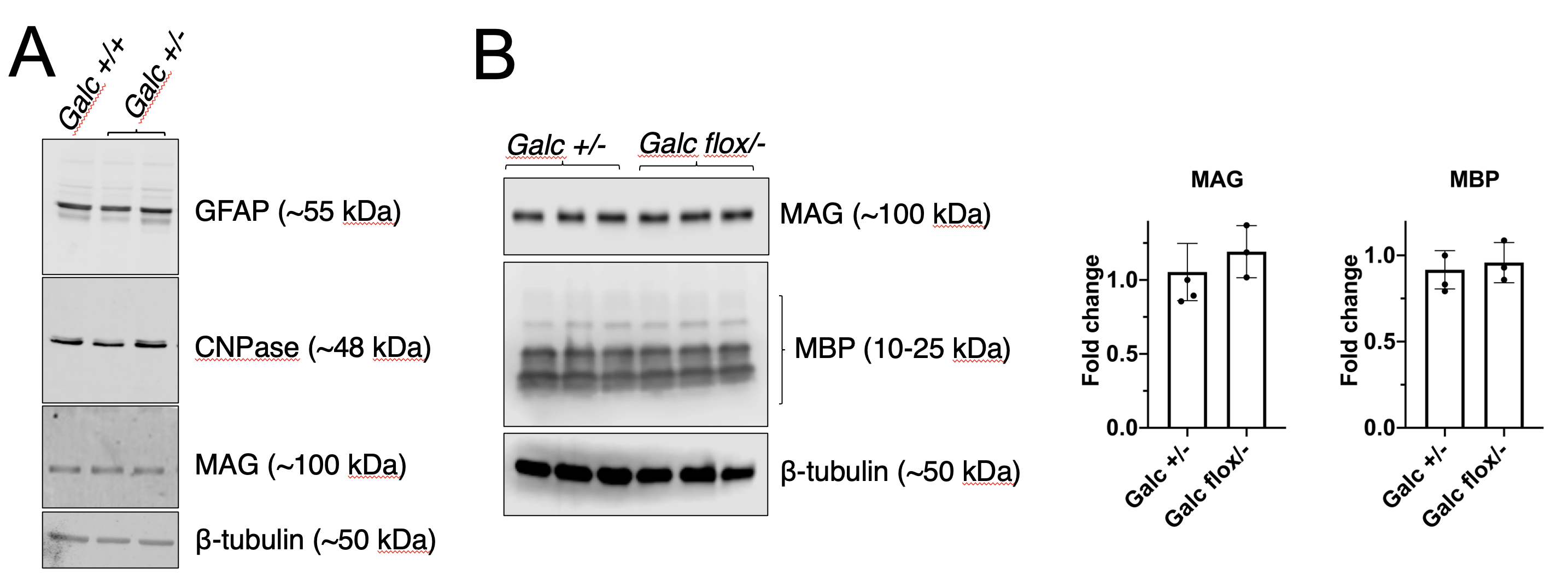

Supplement: S6 Fig — (A) Western blot analysis of total brain extracts shows that the haplodeficient Galc +/− did not affect the levels of myelin proteins and GFAP, compared to Galc +/+ WT. (B) The expression of myelin proteins such as MAG and MBP was not different between both haplodeficient Galc +/− and Galc flox/−. N = 3. The underlying data for B can be found in S1 Data. GALC, galactosylceramidase; MAG, myelin-associated glycoprotein; MBP, myelin basic protein; WT, wild-type. (TIFF) [file pbio.3001661.s006.tiff]
