# Supplementary material for: Neuron-specific ablation of the Krabbe disease gene galactosylceramidase in mice results in neurodegeneration
Source: PLoS Biol. 2022 Jul 5;20(7):e3001661. doi: 10.1371/journal.pbio.3001661 (PMC9255775; doi:10.1371/journal.pbio.3001661)

Figure 6C, 7F * All images were captured by c-digit (Licor) with Bio-rad ECL

Beta-tubulin

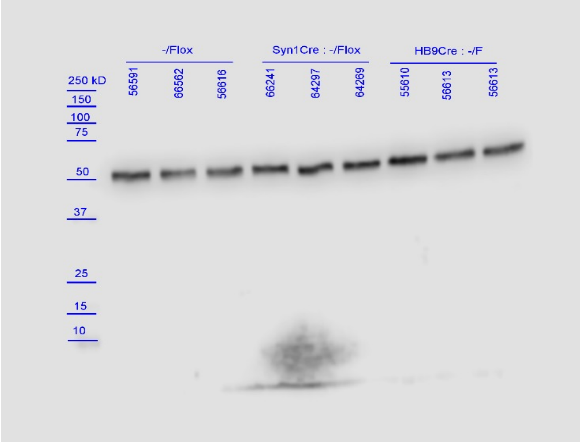

GFAP

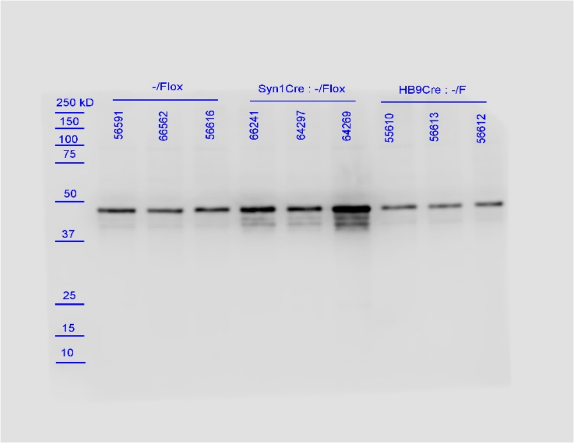

MAG

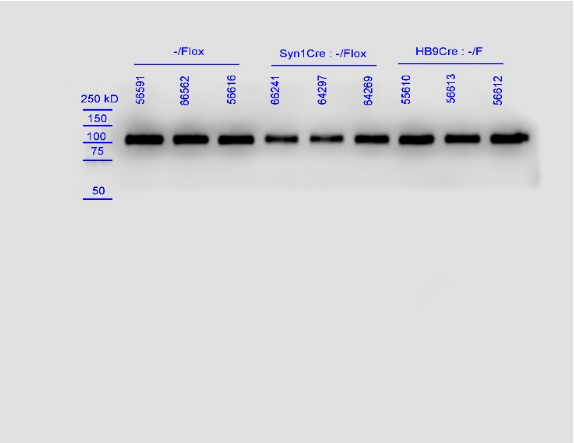

MBP

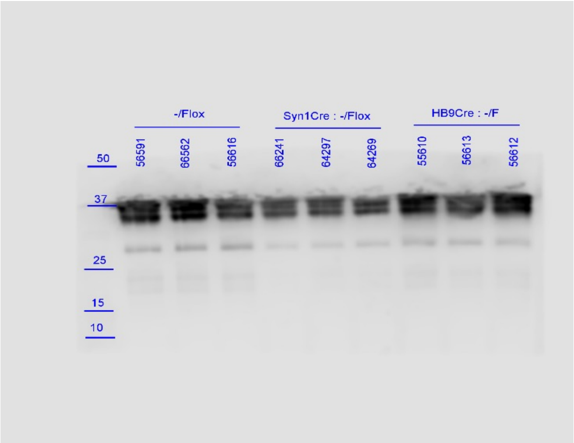

Figure S6A * All images were captured by c-digit (Licor) with Bio-rad ECL

Beta-tubulin

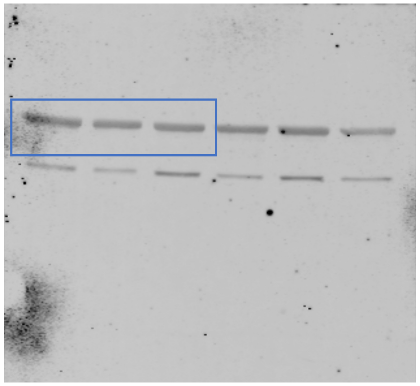

GFAP

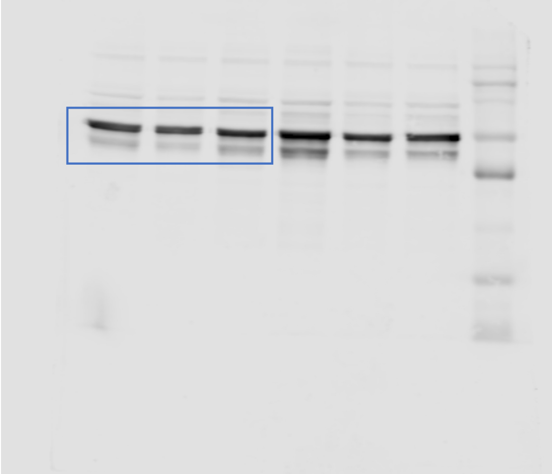

CNPase

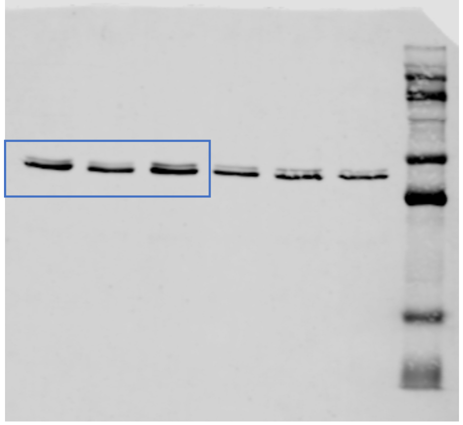

MAG

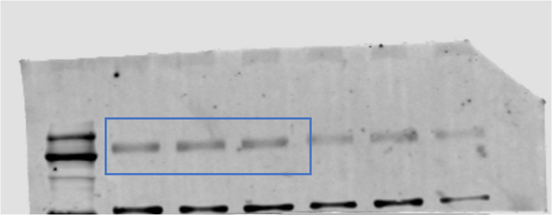

Figure S6B

* All images were captured by c-digit (Licor) with Bio-rad ECL

MAG

MBP

Beta-tubulin

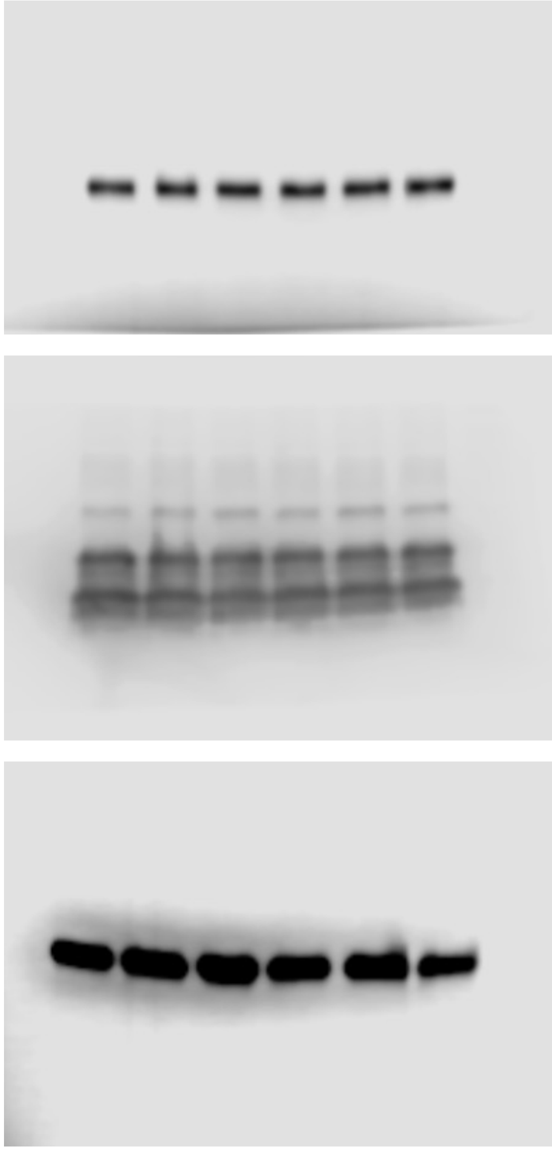

Supplement: S1 Raw images — (PDF) [file pbio.3001661.s008.pdf]
